# D-3-Phosphoglycerate Dehydrogenase

**DOI:** 10.3389/fmolb.2018.00110

**Published:** 2018-12-13

**Authors:** Gregory A. Grant

**Affiliations:** ^1^Departments of Developmental Biology and Medicine, Washington University School of Medicine, St. Louis, MO, United States; ^2^Department of Medicine, Washington University School of Medicine, St. Louis, MO, United States

**Keywords:** d-serine, l-serine, phosphoglycerate, dehydrogenase, biosynthesis

## Abstract

l-Serine is the immediate precursor of d-serine, a major agonist of the N-methyl-d-aspartate (NMDA) receptor. l-Serine is a pivotal amino acid since it serves as a precursor to a large number of essential metabolites besides d-serine. In all non-photosynthetic organisms, including mammals, a major source of l-serine is the phosphorylated pathway of l-serine biosynthesis. The pathway consists of three enzymes, d-3-phosphoglycerate dehydrogenase (PGDH), phosphoserine amino transferase (PSAT), and l-phosphoserine phosphatase (PSP). PGDH catalyzes the first step in the pathway by converting d-3-phosphoglycerate (PGA), an intermediate in glycolysis, to phosphohydroxypyruvate (PHP) concomitant with the reduction of NAD^+^. In some, but not all organisms, the catalytic activity of PGDH can be regulated by feedback inhibition by l-serine. Three types of PGDH can be distinguished based on their domain structure. Type III PGDHs contain only a nucleotide binding and substrate binding domain. Type II PGDHs contain an additional regulatory domain (ACT domain), and Type I PGDHs contain a fourth domain, termed the ASB domain. There is no consistent pattern of domain content that correlates with organism type, and even when additional domains are present, they are not always functional. PGDH deficiency results in metabolic defects of the nervous system whose systems range from microcephaly at birth, seizures, and psychomotor retardation. Although deficiency of any of the pathway enzymes have similar outcomes, PGDH deficiency is predominant. Dietary or intravenous supplementation with l-serine is effective in controlling seizures but has little effect on psychomotor development. An increase in PGDH levels, due to overexpression, is also associated with a wide array of cancers. In culture, PGDH is required for tumor cell proliferation, but extracellular l-serine is not able to support cell proliferation. This has led to the hypothesis that the pathway is performing some function related to tumor growth other than supplying l-serine. The most well-studied PGDHs are bacterial, primarily from *Escherichia coli* and *Mycobacterium tuberculosis*, perhaps because they have been of most interest mechanistically. However, the relatively recent association of PGDH with neuronal defects and human cancers has provoked renewed interest in human PGDH.

## Introduction

**Figure 1:**
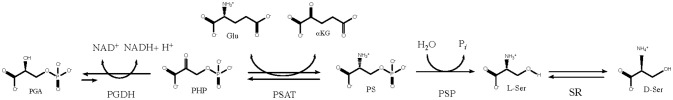
The l-serine biosynthetic pathway uses d-3 phosphoglycerate from glycolysis in the first step. d-serine is produced from l-serine by serine racemase.

d-Serine is an agonist of the n-methyl-d-aspartate (NMDA) receptor and it is synthesized from l-serine by serine racemase (SR) (Fuchs et al., 2006; Ehmsen et al., 2013; Abe et al., 2014). For nearly all organisms, including mammals, l-serine is described as a non-essential amino acid because it is not required in the diet, but is produced by a biosynthetic pathway (Figure 1) (Sallach, 1956; Greenberg and Ichihara, 1957; Hanford and Davies, 1958; Willis and Sallach, 1962, 1964; Walsh and Sallach, 1966; Cheung et al., 1968; Nelson et al., 2009; Voet and Voet, 2011). In these organisms, l-serine is made from the glycolytic intermediate d-3-phosphoglycerate (PGA) and the first enzyme in the l-serine biosynthetic pathway is d-3-phosphoglycerate dehydrogenase (PGDH). It converts PGA to phosphohydroxypyruvate (PHP) with the concomitant reduction of NAD^+^ to NADH. To complete the pathway, phosphoserine amino transferase (PSAT) converts PHP to l-phosphoserine (PS) with the concomitant conversion of glutamate to α-ketoglutarate (αKG), followed by the conversion of PS to l-serine with the loss of phosphate by phosphoserine phosphatase (PSP). The official designation of the genes coding for enzymes in this pathway differ with species. For instance, in *Escherichia coli, Mycobacterium tuberculosis*, and *Homo sapiens*, the gene coding for PGDH is designated as *serA1, Rv2996c*, and *PHGDH*, respectively. In this review, the designation “PGDH” will be used to refer to this enzyme from any species.

The use of the term “non-essential” to describe l-serine is unfortunate because it is a very important amino acid and is very essential as the precursor to many metabolites. In addition to being a precursor for d-Serine, l-serine is also a precursor for the production of such metabolites as glycine, cysteine, tryptophan, phosphatidyl l-serine, sphingolipids, purines, porphyrins, glyoxalate, and glycine (Nelson et al., 2009; Voet and Voet, 2011). As the major precursor to glycine, it also contributes the one-carbon unit (C1) that is the donor in methylation reactions mediated by derivatives of tetrahydrofolate and S-adenosyl methionine.

l-serine can also be produced from glycine by the action of serine hydroxymethyl transferase (SHMT) (Nelson et al., 2009; Voet and Voet, 2011). However, this is not a usual or major route to the production of l-serine because it depletes glycine levels as well as methylene tetrahydrofolate which is the source of one-carbon units used for methylation reactions. Many organisms, including mammals, also contain a pathway that is often mistaken for a second pathway for the production of l-serine. This so-called “un-phosphorylated” pathway actually functions in l-serine degradation (Snell, 1986). The enzyme that is a counterpart to PGDH in this pathway is a d-glycerate dehydrogenase (GDH usually designated *serA2*) and is located in the mitochondria in mammals (Snell, 1975) and the peroxisomes in plants (Voet and Voet, 2011). The existence of a catabolic pathway is necessary because the de-phosphorylation of PS to produce l-serine is irreversible. The “phosphorylated” pathway, located in the cytosol and utilizing PGDH, is therefore the only anabolic source of l-serine in non-photosynthetic organisms. In addition to being a precursor for many other metabolites, l-serine, particularly when present at high levels, can also be converted into pyruvate and ammonia by l-serine dehydratase (LSD). The glycolytic precursor of l-serine, PGA can be generated from glucose by glycolysis or from pyruvate by an abbreviated gluconeogenesis pathway that produces phosphoenolpyruvate and then PGA. In *E. coli*, ~15% of the carbon assimilated when it is grown on glucose passes through l-serine before incorporation into biosynthetic products (Pizer and Potochny, 1964). In humans, ~75% of the l-serine that appears during fasting comes from *de novo* serine synthesis (Kalhan and Hanson, 2012). In mammals, under normal dietary conditions, most of the l-serine is synthesized in the kidney. However, when dietary protein is limiting, a marked increase in l-serine synthesis occurs in the liver (Kalhan and Hanson, 2012). In the central nervous system, l-serine is predominately synthesized in astrocytes rather than neurons (Tabatabaie et al., 2010).

From a structural and mechanistic point of view, the most studied PGDH is that from *E. coli* (Pizer, 1963; Pizer and Potochny, 1964; Rosenbloom et al., 1968; Sugimoto and Pizer, 1968a,b; Winicov and Pizer, 1974; Dubrow and Pizer, 1977a,b; McKitrick and Pizer, 1980; Tobey and Grant, 1986; Schuller et al., 1995; Al-Rabiee et al., 1996a,b; Grant et al., 1996, 1999a,b, 2000a,b, 2001a,b, 2002, 2003, 2004, 2005; Zhao and Winkler, 1996; Grant and Xu, 1998; Bell et al., 2002, 2004; Grant, 2004, 2011, 2012, 2018; Thompson et al., 2005; Dey et al., 2007; Burton et al., 2008, 2009a), followed by that from *M. tuberculosis* (Grant et al., 1999c; Dey et al., 2005a,b, 2008; Burton et al., 2007, 2009b; Xu and Grant, 2014; Xu et al., 2015). There are also reports from various animal tissues (Pizer, 1964; Walsh and Sallach, 1965; Cheung et al., 1969; Pizer and Sugimoto, 1971; Grant and Bradshaw, 1978; Grant et al., 1978; Lund et al., 1986; Fell and Snell, 1988; Achouri et al., 1997), other eukaryotes (Ulane and Ogur, 1972; Ali et al., 2004; Singh et al., 2014), other bacteria (Umbarger and Umbarger, 1962; Umbarger et al., 1963; Saski and Pizer, 1975; Peters-Wendisch et al., 2002, 2005), and plants (Hanford and Davies, 1958; Cheung et al., 1968; Slaughter and Davies, 1968a,b; Rosenblum and Sallach, 1970). More recently, investigations of PGDH from another bacterial species (Zhang et al., 2017) and humans (Grant, 2012; Fan et al., 2015; Xu et al., 2015; Unterlass et al., 2017) have been reported. PGDH has also been implicated in abnormal neural development in humans and as a potential cancer therapy target. These topics will be referenced and discussed later in this review.

## PGDH Types

**Figure 2:**
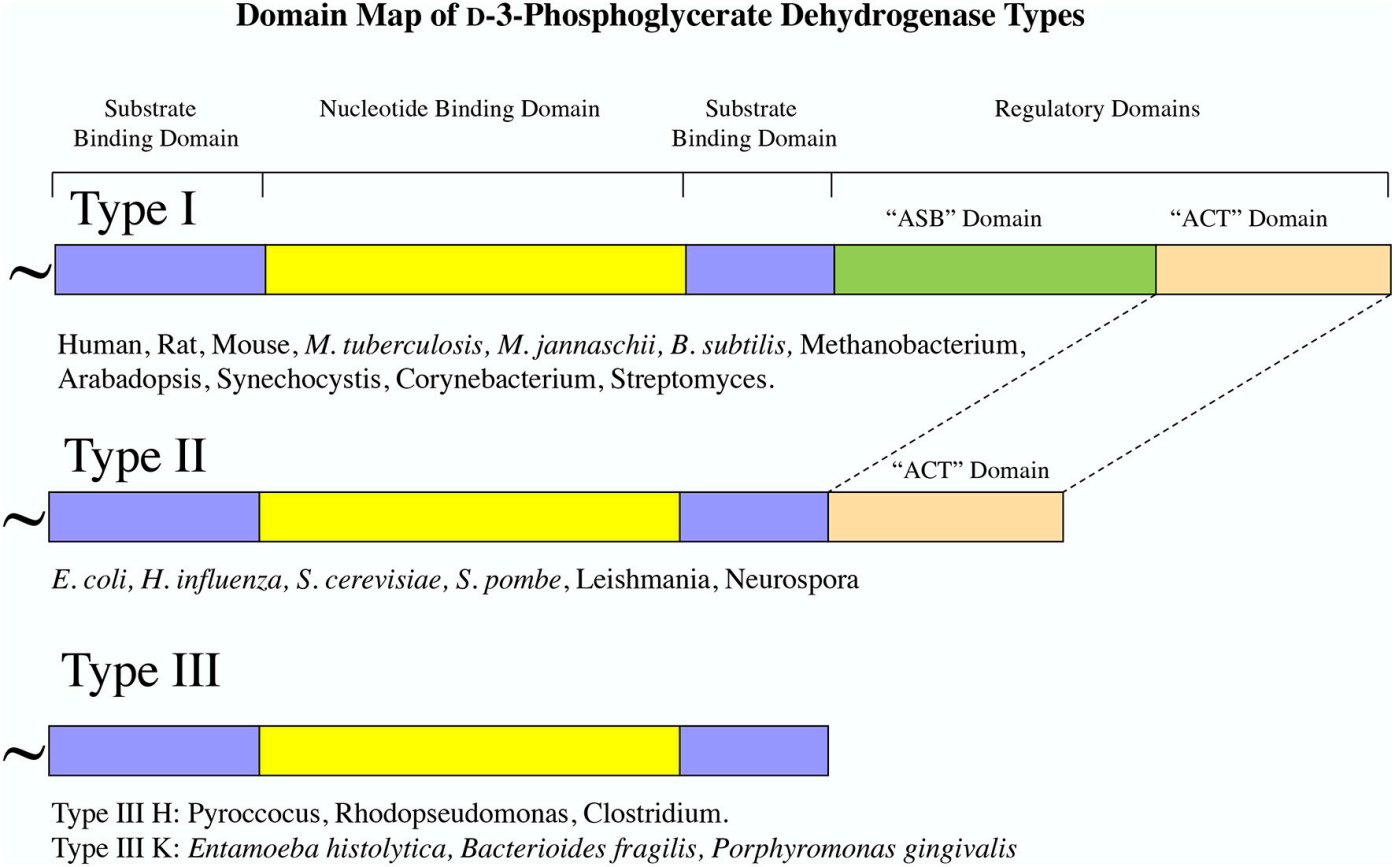
PGDH exists as three different types distinguished by domain structure. The domain structure, going from amino terminus to carboxyl terminus, is illustrated for each type along with representative species containing each type. The “~” symbol signifies that the enzyme from different species can contain different lengths of amino acid sequence at the amino terminus.

**Figure 3:**
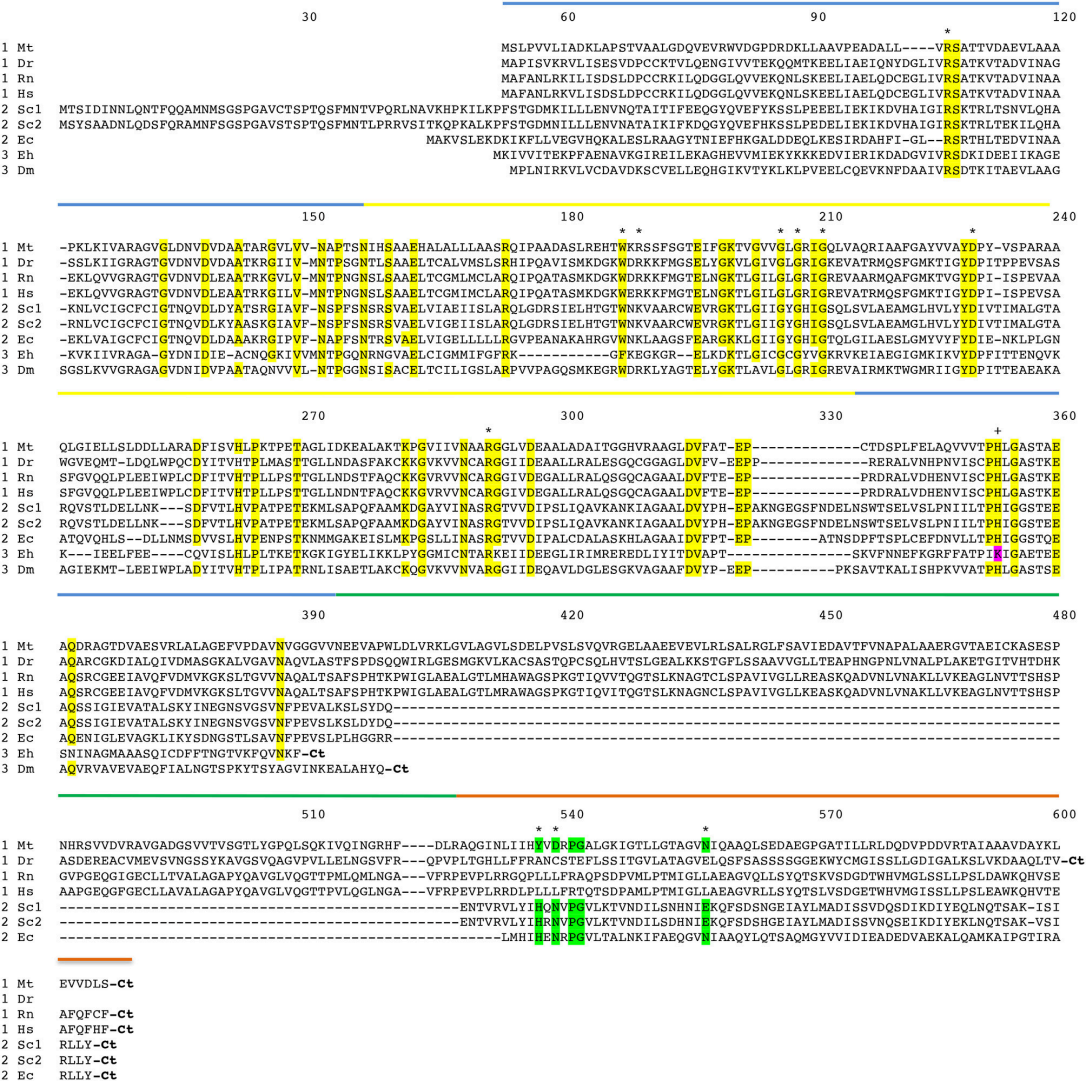
Amino acid sequence alignment of PGDH form representative species. Mt, *Mycobacterium tuberculosis*; Dr, *Danio rerio* (zebrafish); Rn, *Rattus norvegicus*; Hs, *Homo sapiens*; Sc1 and Sc2, two variants from *Saccharomyces cervisiae*; Ec, *Escherichia coli*; Eh, *Entamoeba hystolytica*; Dm, *Drosophila melanogaster*. The numbering designates residue position in the figure rather than the sequence of a particular PGDH. The PGDH type is shown as a numeral in front of the species abbreviation. The domains are highlighted with colored lines. Substrate binding domain, blue; nucleotide binding domain, yellow; ASB domain, green; and ACT domain, orange. Conserved residues are highlighted in yellow. Residues involved in l-serine binding at the ACT site are highlighted in green. The active site lysine in some type 3 enzymes is highlighted in magenta. Asterisks designate residues involved in substrate and effector binding and the plus sign identifies the active site histidine or lysine. The carboxyl termini are designated “Ct”.

Although all PGDH enzymes (EC 1.1.1.95) catalyze the same reaction, they exhibit certain mechanistic differences and they can be divided into three structural types based on domain structure (Grant, 2012) (Figure 2). Type 1 enzymes are composed of four domains, the substrate binding domain, the nucleotide binding domain, the ASB domain (where ASB stands for allosteric substrate binding), and the regulatory domain which is an ACT domain (Aravind and Koonin, 1999; Grant, 2006) (where ACT stands for the first letters in Aspartate kinase, Chorismate mutase, and TyrA). As will be discussed later, the regulatory domain designation is based on its role in the regulation of enzyme activity by l-serine. Although it is often reported, particularly in introductory textbooks, that PGDH in general is feedback inhibited by l-serine (Walsh and Sallach, 1965; Slaughter and Davies, 1968a; Rosenblum and Sallach, 1970; Fell and Snell, 1988; Achouri et al., 1997), all mammalian enzymes so far studied as well as those from many other species have lost this ability. The ASB domain is so named because it functions as a substrate binding regulatory site in PGDH from some species (Dey et al., 2005a; Burton et al., 2007, 2009b). The function of the other two domains corresponds to their designation, namely that they function mainly in the binding of substrate and coenzyme. Type 2 enzymes are missing the ASB domain and consist of a substrate binding domain, a nucleotide binding domain, and a regulatory (ACT) domain (Schuller et al., 1995). Type 3 enzymes consist of only a substrate binding domain and a nucleotide binding domain (Ali et al., 2004). In addition, as demonstrated in Figures 2, 3, some, but not all, Type 3 enzymes utilize lysine as the catalytic residue (Ali et al., 2004; Singh et al., 2014) instead of the histidine that is found in all other PGDH types. Interestingly, there does not appear to be a pattern of domain type associated with type of organism. For instance, Type 1 enzymes containing all four domains are found in mammals, plants, and bacteria and Type 2 enzymes are found in eukaryotic organisms as well as bacteria. It is important to note that the presence of a homologous structural domain does not necessarily mean that it is functional.

## PGDH Homology

The amino acid sequence alignment of representative PGDHs is shown in Figure 3. The numbering refers to position in the figure rather than in any particular sequence. The types are shown before the species abbreviation and the domains are highlighted with colored lines. Residues known to be involved in catalysis or ligand binding are shown with an asterisk or a plus sign (see Figure legend). The arginine residues at positions 106 and 189 are involved in interaction with the substrate phosphoryl group. The aromatic amino acid at position 187 forms the bottom of the active site cleft. The glycine residues at positions 205, 207, and 210 and the aspartic acid residue at position 230 are conserved residues in the Rossman fold involved in coenzyme binding. The arginine residue at position 291 anchors the substrate into the active site by interaction with the substrate carboxyl group. The active site histidine (or lysine) that donates a proton is at position 353. The residues that participate in binding l-serine at the regulatory site are at positions 537, 539, and 557.

A comparison of the conserved amino acid residues shows that there is a relatively low degree of identity between human PGDH and those of the non-mammalian PGDHs. There is 36.8, 31.9, and 24.5% identity of the nucleotide binding and substrate binding domains combined, between Human and *M. tuberculosis, E. coli*, and *Entamoeba histolytica*, respectively. There is 30.7, 27.6, and 24.5% identity of all common domains between Human and *M. tuberculosis, E. coli*, and *E. histolytica*, respectively. There is only 8.4% identity of the ASB domains between Human and *M. tuberculosis*, and 3.9 and 8.2% identity of the ACT domains between Human and *M. tuberculosis, E. coli*, respectively. Among mammalian species, such as between human and rat PGDH, there is a 94.6% identity.

## PGDH Structure

### Oligomeric Conformations

**Figure 4:**
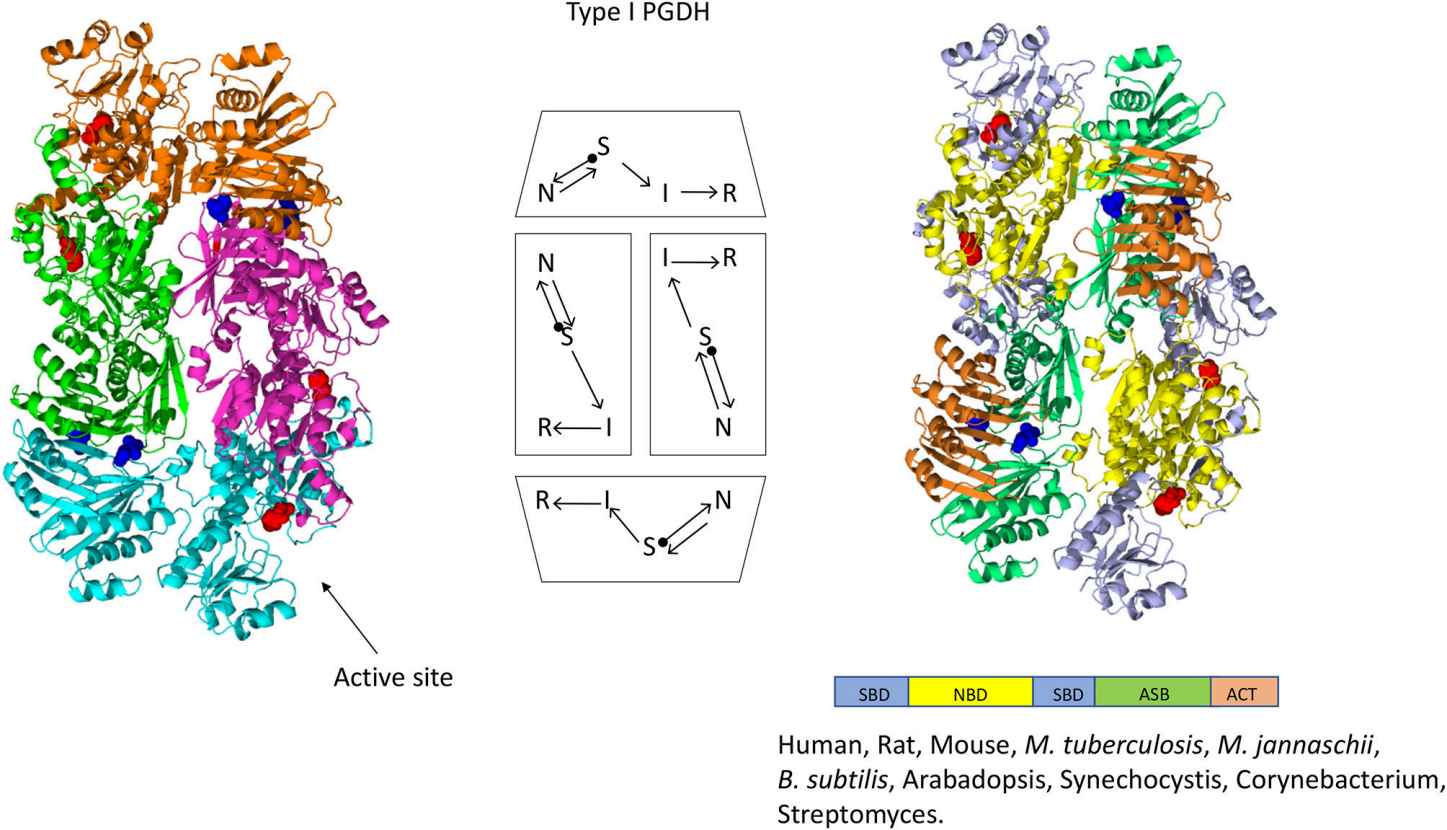
Crystal structure of a Type I PGDH from *M. tuberculosis*. On the left, each subunit is colored differently. In the center is a diagram of the subunits showing the course of the polypeptide chain from amino terminus, designated by a black dot, to the carboxyl terminus. On the right, the individual domains are colored according to the scheme shown below the structure. Representative species with Type 1 enzymes are listed.

**Figure 5:**
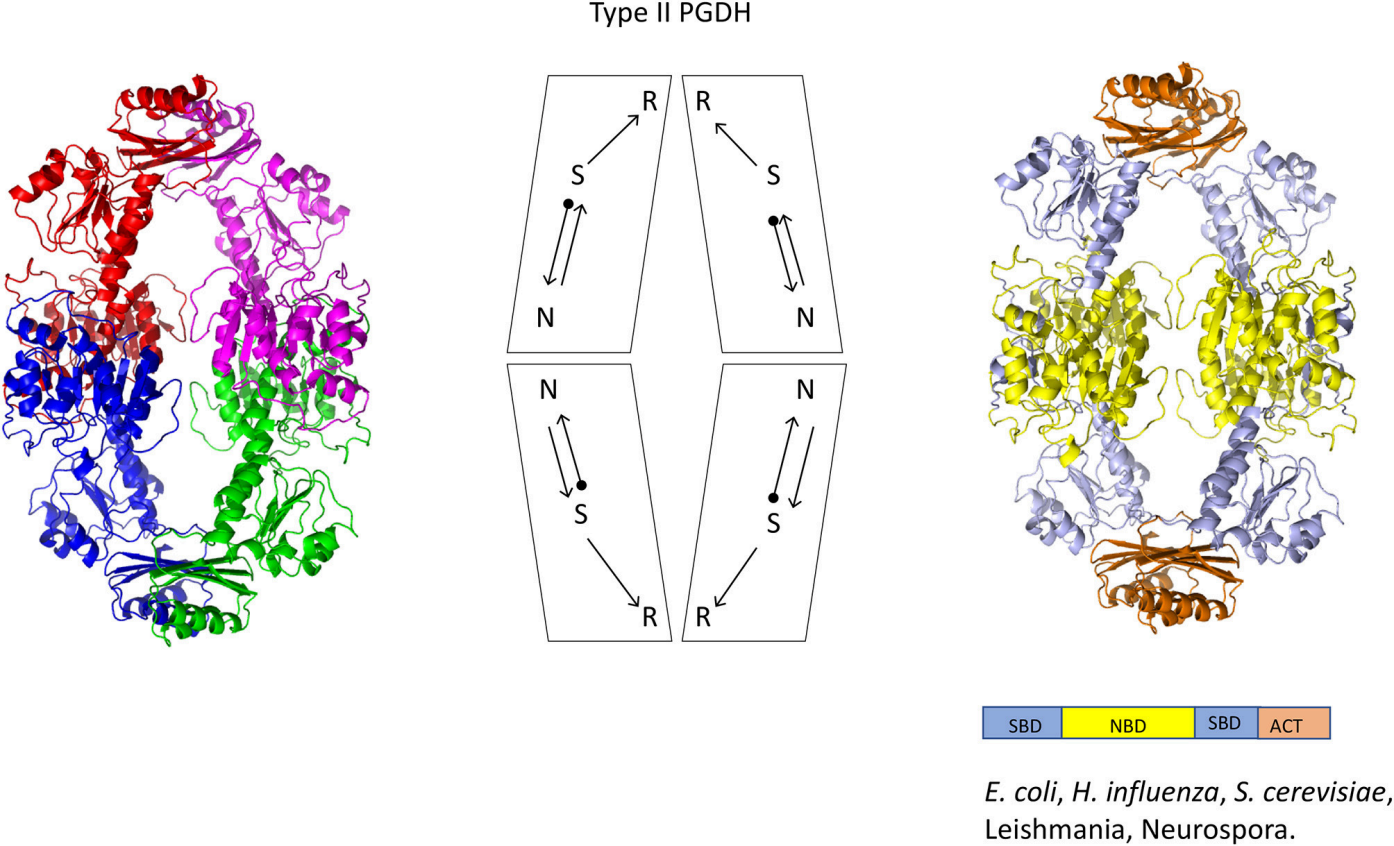
Crystal structure of a Type II PGDH from *E. coli*. On the left, each subunit is colored differently. In the center is a diagram of the subunits showing the course of the polypeptide chain from amino terminus, designated by a black dot, to the carboxyl terminus. On the right, the individual domains are colored according to the scheme shown below the structure. Representative species with Type II enzymes are listed.

**Figure 6:**
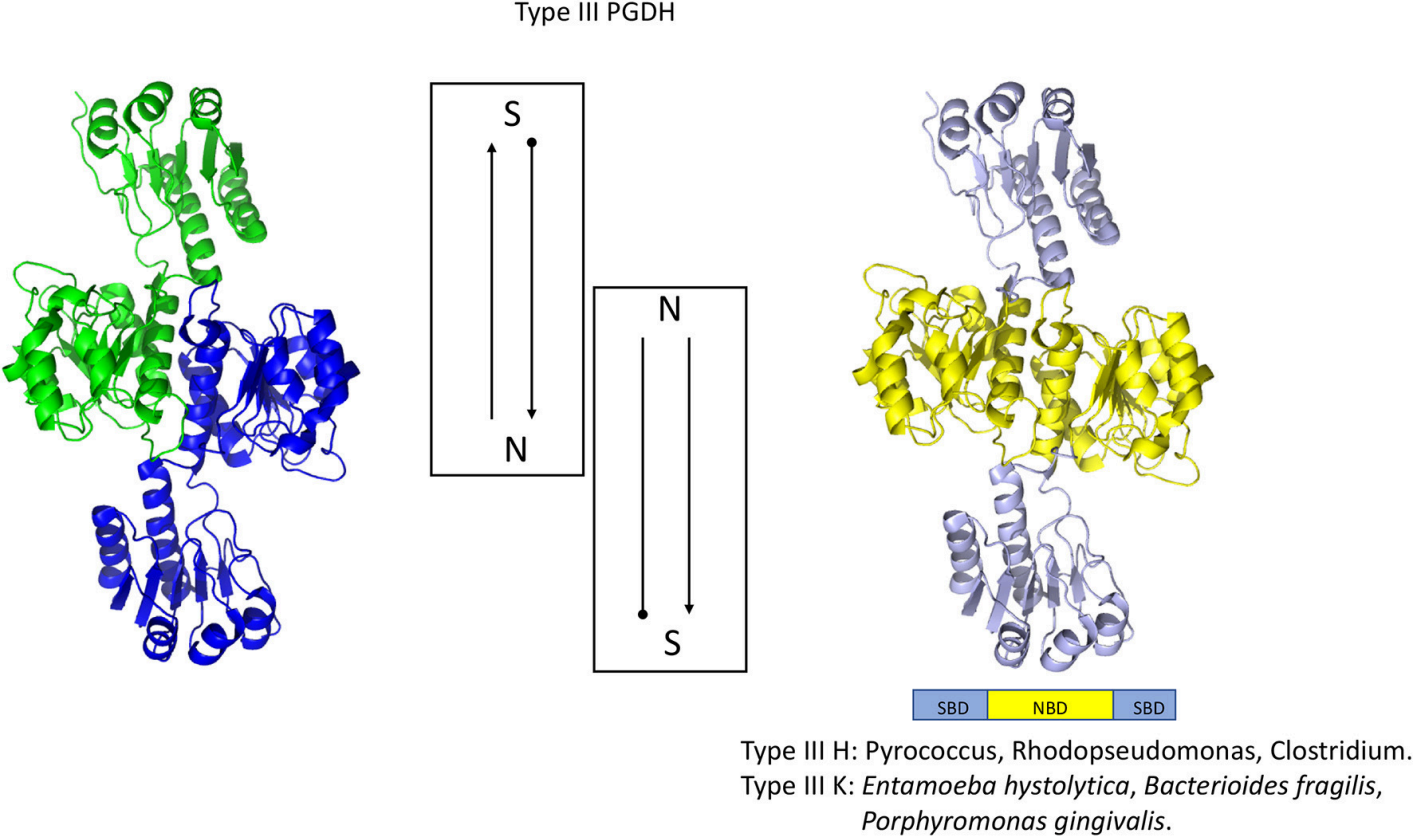
Crystal structure of a Type III PGDH from *E. hystolytica*. On the left, each subunit is colored differently. In the center is a diagram of the subunits showing the course of the polypeptide chain from amino terminus, designated by a black dot, to the carboxyl terminus. On the right, the individual domains are colored according to the scheme shown below the structure. Representative species with Type III enzymes are listed.

The structure of PGDH differs depending on its domain makeup or Type (Figures 4–6). The only complete PGDH structures that have been determined and published are a Type I from *M. tuberculosis* (Dey et al., 2005a, Figure 4), a Type II from *E. coli* (Schuller et al., 1995, Figure 5), and a Type III from *E. histolytica* (Singh et al., 2014, Figure 6). A partial structure of human PGDH has been published (Unterlass et al., 2017) showing the substrate and nucleotide binding domains but without the ASB and ACT domains which were proteolytically removed to facilitate crystallization. The structure of a Type II PGDH from *Brucella melitensis* and Type III PGDHs from *Lactobacillus plantarum, Pyrococcus horokoshi, Sulfolobus tokodaii, Ralstonia solanacearum*, and *Vibrio cholera* have been deposited in the RCSB protein data bank but have not been described in a publication.

**Figure 7:**
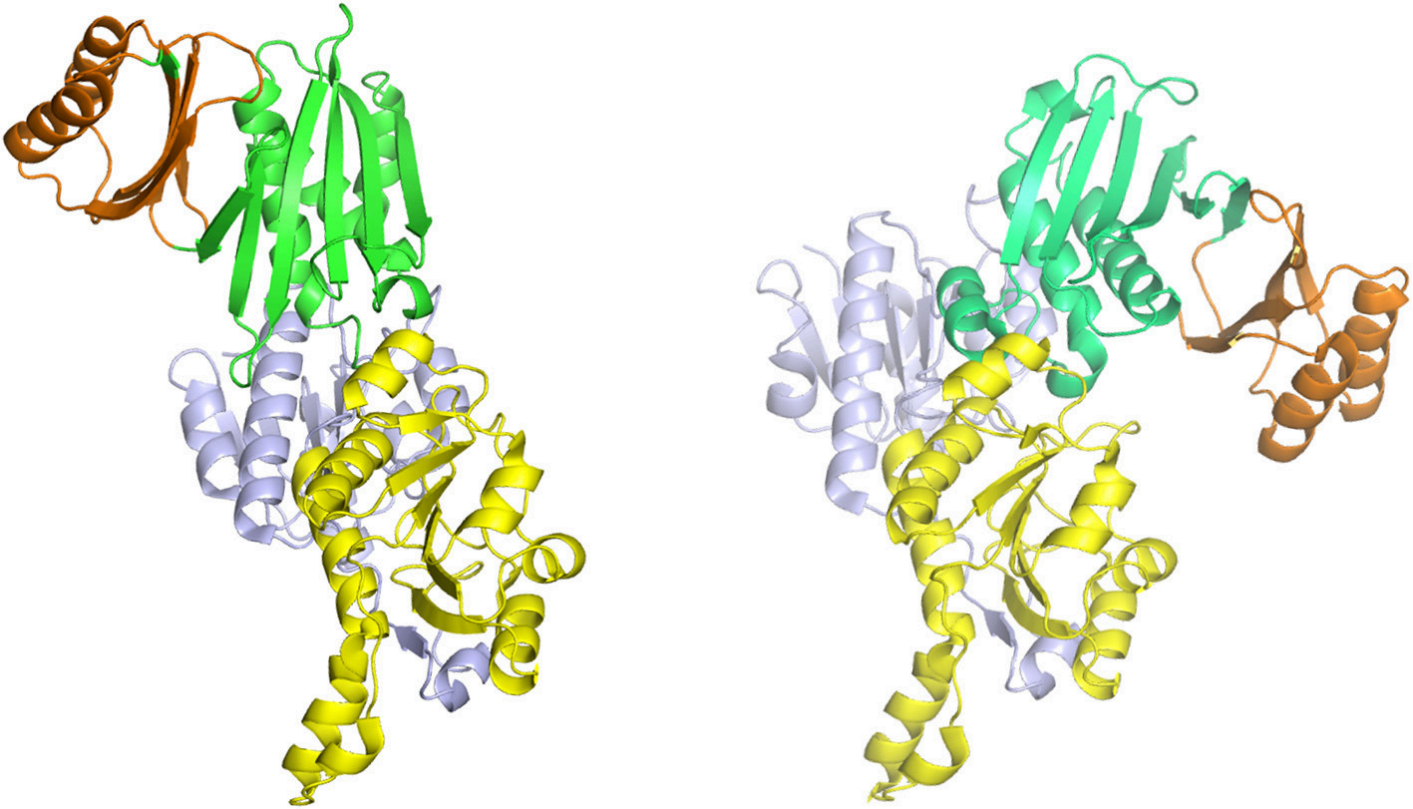
The syn- **(right)** and anti- **(left)** configurations of the subunits of *M. tuberculosis* PGDH. The subunits are pictured with their substrate (blue) and nucleotide (yellow) binding domains in essentially the same orientation. The ASB (green) and ACT (orange) domains are rotated to the left in the anti-configuration and to the right in the syn-configuration.

The one structure of a Type I PGDH that has been determined is from *M. tuberculosis* (Figure 4). It is a tetramer with identical subunits with a molecular weight of 54,522. However, the subunits are identical with respect to amino acid composition but not with respect to domain orientation (Dey et al., 2005a). Interestingly, the subunits adopt two different conformations, designated syn- and anti- (Figure 7), arranged in the tetramer as shown in Figure 4 and which results in an internal asymmetry. The syn- and anti-conformations differ by a rotation of ~180° at a position composed of three consecutive glycine residues (position 389–391 in Figure 3). This results in two different sets of inter-subunit interactions between the nucleotide binding and ASB domains resulting in different portions of the respective subunits being exposed to solvent. A diagram of the subunit domain orientations is provided in Figure 4 showing that the two syn-conformers are in the middle and the two anti-conformers are at the ends of the elongated tetramer.

*M. tuberculosis* PGDH has also been shown to exhibit multiple oligomeric equilibrium states (Xu and Grant, 2014) modulated by phosphate and polyphosphates (Figure S1). In the absence of these ions, the enzyme is in equilibrium between an inactive dimer and an active tetramer that is relatively insensitive to inhibition by l-serine. However, in the presence of phosphate ion, a conversion to active tetramers and active octamers occurs. These two species are in equilibrium and both are very sensitive to inhibition by l-serine. Small polyphosphates, such as pyrophosphate and triphosphate, induce a conversion to an active dimer that is insensitive to l-serine. A similar dependency on phosphate ion for oligomeric state is also observed for human PGDH (unpublished) which is also a Type I enzyme.

The dependency of activity on phosphate ion concentration is further illustrated when the activity of *M. tuberculosis* PGDH is monitored as a function of time (Dey et al., 2005b, Figure S2). When the enzyme is diluted 500-fold in the presence of 200 mM KPO_4_ buffer, the enzyme retains activity. However, a 500-fold dilution in 20 mM KPO_4_ buffer results in a time dependent loss of activity that fits best to a double exponential function. This demonstrates that there are at least two enzyme forms that lose activity with discernable dissociation constants. These forms most likely correlate with the observed changes in oligomeric state.

The mammalian enzymes are also Type I PGDHs. The subunit molecular weight of human PGDH is 56,651, but it has been crystallized only after removal of the ASB and ACT domains. Therefore, it is not known if mammalian PGDHs adopt the same internal asymmetry of the tetramer found in the *M. tuberculosis* PGDH. Since they do not contain the triple glycine sequence that presumably allows for the domain rotation found in *M. tuberculosis* PGDH, it is possible that they do not share the same tetrameric configuration. Furthermore, it is not known whether *M. tuberculosis* PGDH actually adopts the asymmetric conformation in solution. Since *M. tuberculosis* PGDH could only be crystallized in the presence of 1 M tartrate, and a tartrate molecule is found between adjacent ASB domains, it has been speculated that the crystal structure may represent only one of at least two alternative conformations and that these alternative conformations may be responsible for the peculiar phosphate dependent sensitivity to l-serine (see section Feedback Inhibition) and the variations in oligomeric states.

A representative structure of a Type II PGDH is from *E. coli* (Figure 5). It is a tetramer of identical subunits with respect to amino acid composition and domain orientation with a subunit molecular weight of 44,044. The tetramer is a dimer of dimers, with one dimer interface at the nucleotide binding domains and the other at the ACT or regulatory domains.

A representative structure of a Type III PGDH is from *E. histolytica* (Figure 6). Since it lacks ASB and ACT domains, the only subunit interface is at the catalytic domains resulting in a dimeric configuration. Its subunit molecular weight is 33,469.

### Catalytic Sites

**Figure 8:**
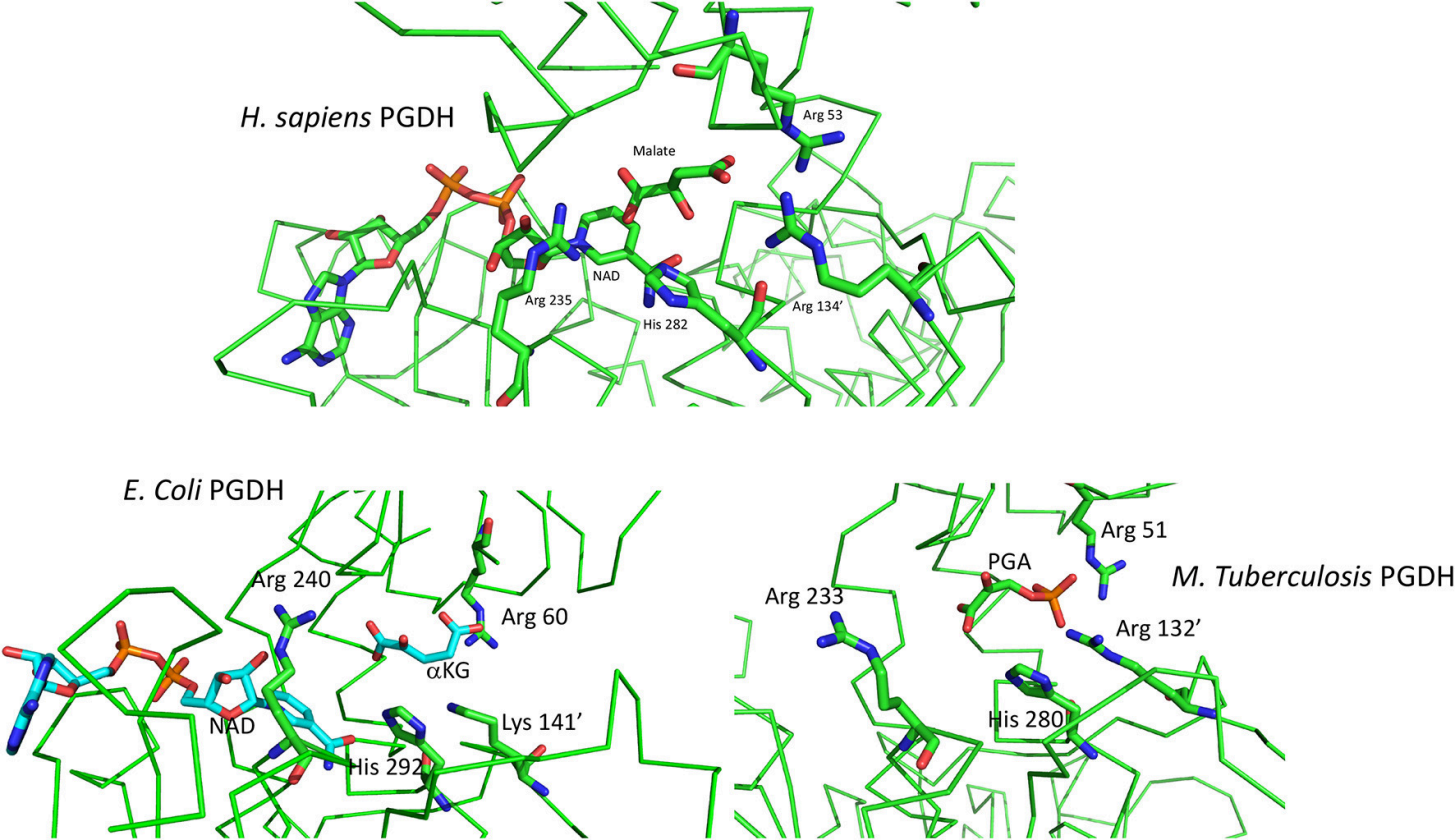
**(Top)** Depiction of the active site of human PGDH from pdb 2g76 with malate and NAD bound. Some of the alpha chain in the background has been cut away for clarity. **(Bottom)** Depiction of the active site of *E. coli* PGDH from pdb 1yba (left) and of *M. tuberculosis* PGDH from pdb 2ddn (right).

The basic mechanism of all PGDHs is the same. The substrate, PGA, is oxidized by the transfer of a proton to the active site histidine or lysine and a hydride ion to NAD^+^, to yield PHP. The catalytic site of human PGDH with NAD and malate bound is depicted in Figure 8. The three cationic residues that interact with the acidic ends of the substrate are shown and are common to all PGDH active sites. In Figure 3, Arg 53 corresponds to position 107, Arg 134′ to position 189, Arg 235 to position 291, and His 282, which is the proton donor, to position 353. Arg 235 interacts with the carboxyl of PGA/PHP while the other two arginine residues interact with the phosphate group. In this depiction, the active site is closed, but since malate is a shortened version of the substrate, the spatial configuration of the residues is not optimal. The active sites in the crystal structures of *E. coli* and *M. tuberculosis* PGDH (Figure 8) are in a more open configuration, as if they are poised just prior to closing of the active site cleft. The open conformation of the active site may be due to crystal packing constraints that aren't in play with the human structure since the ASB and ACT domains are missing in the crystallized form reported. In *M. tuberculosis* PGDH, Arg 51 corresponds to position 107, Arg 132′ to position 189, Arg 233 to position 291, and His 280 to position 353.

The order of substrate and coenzyme binding in *E. coli* PGDH (Grant et al., 2002) and *Pseudomonas* species PGDH (Zhang et al., 2017) is coenzyme before substrate. This is also apparently the case for human PGDH (Unterlass et al., 2017) since the crystal structure contains NADH (Unterlass et al., 2017). However, this has not been determined conclusively from kinetic analysis of the complete enzyme. The order in *M. tuberculosis* PGDH is substrate before coenzyme, opposite of that for the others. The crystal structures of *M. tuberculosis* PGDH show that the coenzyme sites have restricted access to solvent. In the syn-conformation, the coenzyme site is covered by the ASB domain, resulting in a narrow channel leading to solvent. In the anti-conformation, the ASB is rotated away from the coenzyme site but is blocked by a long flexible loop. Therefore, significant domain and loop movements are likely related to coenzyme binding in solution. Superimposing the human and *M. tuberculosis* PGDH catalytic sites shows that the latter has a more open cleft, requiring a rotation for closure, but the PHP is in approximately the same position as the malate in the human structure. In *E. coli* PGDH, Arg 60 corresponds to position 107, Lys 141′ to position 189, Arg 240 to position 291, and His 292 to position 353. Note that in *E. coli* PGDH a lysine residue is present at position 189 instead of an arginine. NAD is not present in the *M. tuberculosis* structure because it binds after a conformational change induced by substrate binding. For both *E. coli* and *M. tuberculosis* PGDH, a rotation of the substrate binding domain relative to the nucleotide binding domain is necessary to close the cleft and move the various elements into place for catalysis.

### Effector Binding at the ACT and ASB Domain Interfaces

**Figure 9:**
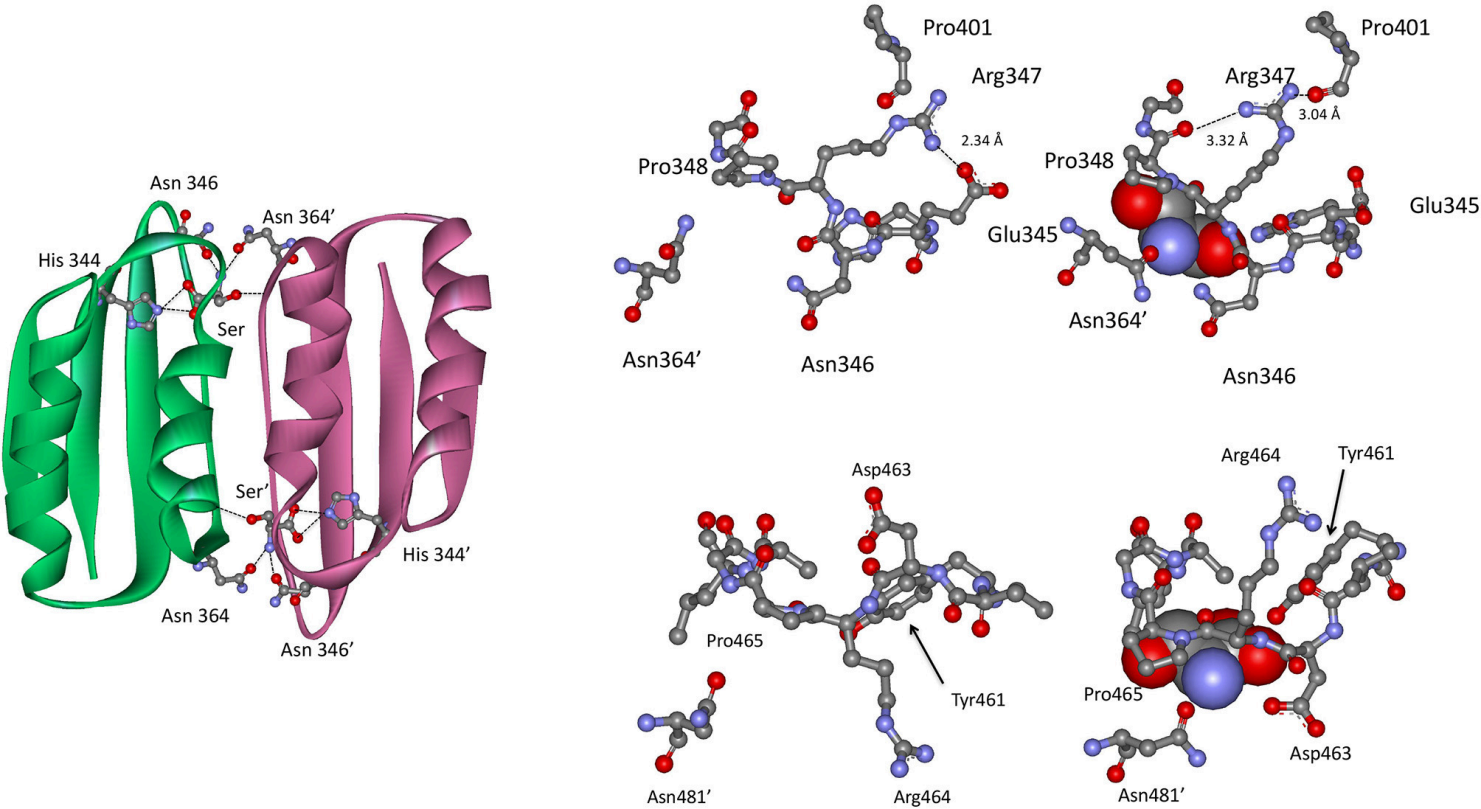
**(Left)** The ACT domain interface in *E. coli* PGDH showing the interaction of l-serine with specific residues. Reprinted from Grant (2012). **(Top Right)** The serine binding site in *E. coli* PGDH. The serine atoms are shown as spheres in the right-hand structure. Reprinted from Grant (2012), with permission from Elsevier © 2011. **(Bottom Left)** The serine binding site in *M. tuberculosis* PGDH. The serine atoms are shown as spheres in the left-hand structure. Reprinted from Grant (2012), with permission from Elsevier © 2011.

In the Type II PGDH from *E. coli*, feedback inhibition of catalytic activity occurs when l-serine binds at the ACT domain interface as shown in Figure 9. The serine carboxyl group hydrogen bonds to the imidazole nitrogen of His 344 (position 537 in Figure 3), and the amino group of serine bonds to the amide side chains of Asn 346 (position 539) and Asn 364′ (position 557). The hydroxyl group of serine does not form hydrogen bonds directly with an amino acid but rather interacts through a water molecule with the main chain carbonyl groups of Thr 352 (position 545) and Val 363′ (position 556). When serine binds, there is a rotation of the substrate binding domain-ACT domain relative to the nucleotide binding domain. This has a significant effect on the conformation of the active site and is likely responsible for the inhibition of activity caused by serine binding. l-serine enters the effector site through an opening at the surface around Asn 364′. Upon binding, the opening is covered by movement of Asn 364′ and Pro 348 (Figure 9). The two binding sites at each ACT domain interface assume different conformations in the absence of serine. One is open and one is closed similar to the structure seen when serine is bound. Since all four sites are occupied in the crystal structure (pdb 1psd), the binding of l-serine to the open site apparently induces the other site to open. Binding studies (Grant et al., 2001b) show that serine binding is positively cooperative for the first two sites in the tetramer, located at opposite ACT domain interfaces, and negatively cooperative for the last two sites to be occupied.

In *M. tuberculosis* PGDH, l-serine is bound at the interface of the ACT domains (Figure 9) as it is in *E. coli* PGDH. However, the interaction with serine is somewhat different. The serine carboxyl group is hydrogen bonded to the hydroxyl group of Tyr 461 (position 537 in Figure 3) and the side chain oxygen of Asp 463 (position 539). The amino group of serine hydrogen bonds to the side chain of Asn 481′ on the opposite subunit (position 557) and the serine hydroxyl is hydrogen bonded directly to main chain amide of Leu 468 (position 544). As is the case with *E. coli*, the pocket closes when serine binds as a result of Asn 481′ moving over the pocket opening. This is accompanied by a movement of Asp 463 and Arg 464 (positions 539 and 540) with Asp 463 forming an additional hydrogen bond with serine.

**Figure 10:**
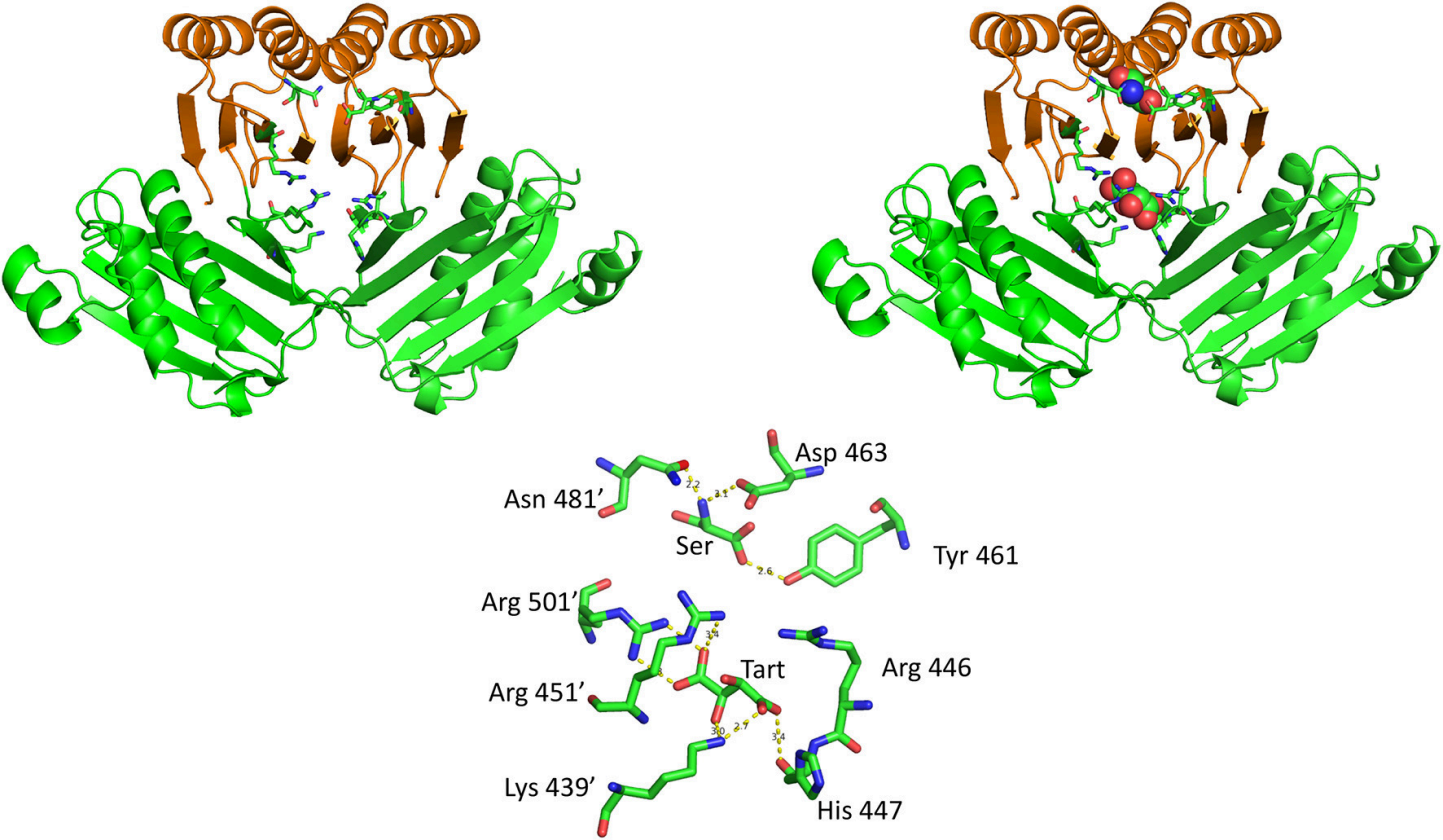
The relationship of the ACT (orange) and ASB (green) domains in *M. tuberculosis* PGDH. The depiction on the left shows the domains in the absence of bound ligands and the depiction on the right shows L-serine and tartrate bound at their respective sites. The stick model at the bottom shows the specific residue interactions seen in the crystal structure.

The crystal structure of *M. tuberculosis* PGDH contains a molecule of tartrate bound at the interface of adjacent ASB domains (Figure 10). This site is populated with a number of cationic residues that interact with the tartrate, including Lys 439, Arg 446, His 447, Arg 451, and Arg 501 (positions 512, 519, 520, 524, and 576, respectively, in Figure 3). Note that Arg 501 is actually in the ACT domain and produces the largest decrease in serine inhibition when it is mutated to alanine (Burton et al., 2009b). Since tartrate is an analog of the substrate, it was originally proposed that this may be an allosteric substrate binding site. Alteration of the residues at the ASB interface by mutagenesis, demonstrated that this site appeared to bind substrate which resulted in an attenuation of catalytic activity (Burton et al., 2009b; Grant, 2012). It has also been proposed, but not definitively shown, that this is also the phosphate binding site and that interplay between substrate and phosphate ion at this site results in phosphate dependent inhibition of catalytic activity when serine binds to the ACT domain (Xu and Grant, 2014).

## Regulation of Catalytic Activity

The production of l-serine is regulated at the level of enzyme activity of PGDH by three main mechanisms: the equilibrium of the reaction, substrate inhibition, and feedback inhibition. However, as noted previously, all three are not necessarily operative in PGDH from all organisms.

### Catalytic Activity and the Equilibrium State of the Reaction

The equilibrium of the reaction catalyzed by PGDH lies far in the direction of PGA, opposite of that leading to serine synthesis (Sugimoto and Pizer, 1968b). At equilibrium, < 5% of the substrates and products are in the form of PHP. The reaction is pulled in the forward direction by the downstream enzymes despite the strong tendency for PGDH to act in the opposite direction. The reaction catalyzed by PSAT is freely reversible, but the de-phosphorylation of l-serine by PSP is not reversible and is the point of no return. This latter reaction, in effect, acts like a sink, keeping serine from being depleted by reaction in the reverse direction and assuring that the flux of the pathway continues in the direction of l-serine synthesis.

As a result of the equilibrium state of the reaction, it is difficult to assay PGDH in the forward direction and historically it has been assayed mostly in the reverse direction using PHP as the substrate. Forward assays using PGA as the substrate usually employ fluorescence monitoring for sensitivity and with hydrazine added, presumably to trap the PHP produced (Sugimoto and Pizer, 1968b), but they are difficult to perform accurately. In order to measure the activity of PGDH in the forward direction, it is best to use a coupled assay that depletes either PHP or NAD^+^ in order to drive the reaction forward. However, as noted below (see section Alternate Substrates), the activity of *E. coli* PGDH cannot be measured by monitoring the formation of NADH when coupled with PSAT because of its conversion back to NAD^+^ by αKG generated by PSAT. This is not the case for *M. tuberculosis* and human PGDH, where the production of NADH can be measured in the coupled reaction. The kinetic constants for PGDH from several species are listed in Table 1.

**Table 1:** Catalytic properties of d-3-phosphoglycerate dehydrogenase^a^.

**Reaction**	***K_***m*,**_*_**substrate**_ (mM)**	***K_***m*,**_*_**coenzyme**_ (mM)**	***k*_**cat**_ (s^**−1**^)**	***k*_**cat**_/*K_***m*,**_*_**substrate**_ (M^**−1**^s^**−1**^)**	**l-Serine IC_**50**_ (Hill coefficient) (μM)**
***E. coli***
PHP reduction^e^	0.0032^b^	< 0.01	28^b^	~9 × 10^6b^	2–13^d^ (~2)
αKG reduction^e^	0.088^b^	< 0.01	12–33^d^	~4 × 10^5b^	2–8^d^
PGA oxidation	1.2^b^	ND^c^	0.6^b^	~5 × 10^2b^	44
HGA oxidation	0.4^b^	ND^c^	0.7^b^	~2 × 10^3b^	36
PGA oxidation^h^ (PGDH/PSAT)	NDS^c^			
***M. tuberculosis***
PHP reduction^e^	0.17	0.06^f^	2,400	~1 × 10^7^	36(~2)
αKG reduction	DNR^c^			
PGA oxidation	ND^c^			
HGA oxidation	ND^c^			
PGA oxidation MOPS^h^	0.54	0.06	1.4	2.6 × 10^3^
(PGDH/PSAT) PO${}_{4}^{\text{f},\text{h}}$	9.2	5.2	3	3.3 × 10^2^	13 (1.5)
**HUMAN**
PHP reduction^e^	0.10	0.02	300	~3 × 10^3^	DNI^c^
KG reduction	DNR^c^			
PGA oxidation^h^ MOPS (PGDH/PSAT)	0.48	0.49	0.4	0.8 × 10^3^	DNI^c^
**RAT**^**e**^
PHP reduction^e^	~0.015^g^	0.025	ND^c^	ND^c^	DNI^c^
PGA oxidation	0.1	0.027	ND^c^	ND^c^	DNI^c^
αKG reduction	DNR^c^			

a *PHP, phosphohydroxypyruvic acid; αKG, α-ketoglutarate; PGA, d-3-phosphoglycerate; HGA, d-hydroxyglutaric acid. Values are per tetramer*.

b *From Zhao and Winkler [29]*.

c *ND, not determined; DNI, does not inhibit; DNR, does not react; NDS, not detectable spectrophotometrically at 340 nm*.

d *Value varies slightly from different groups. Note that in all cases, all of the kinetic parameters can vary depending on the buffer used*.

e *Exhibits substantial substrate inhibition in phosphate buffer, pH 7.0*.

f *Unpublished*.

g *Estimated from Achouri et al. (1997)*.

h *From Grant (2018)*.

### Substrate Inhibition

In addition to the equilibrium of the PGDH catalyzed reaction favoring PGA, human and *M. tuberculosis* PGDH exhibits significant substrate inhibition by PHP (Xu et al., 2015, Figure S3). Therefore, theoretically, if PHP were to accumulate to a certain level, its conversion to PGA would be inhibited, counteracting to some extent the equilibrium tendency of the reaction. However, there are no definitive studies of pathway flux that demonstrate that this is functional physiologically. PHP is inherently unstable and the investigations of metabolite levels that are available (in bacteria, Bennett et al., 2009) don't list PHP as a measurable metabolite. Substrate inhibition by PHP is also less pronounced in phosphate buffer than other buffers that have been reported (Xu et al., 2015).

### Feedback Inhibition

As noted earlier, textbooks often refer to PGDH as being inhibited by the end-product of the pathway, l-serine. While this is true for PGDH from some organisms, it does not pertain to that from all species. When it does occur, it results from the interaction of l-serine with the regulatory or ACT domain. However, the sensitivity of PGDH to l-serine concentration varies greatly. PGDH from *Bacillus subtilis* (Saski and Pizer, 1975) and *Corynebacterium glutamicum* (Peters-Wendisch et al., 2002, 2005) are Type I enzymes that are relatively insensitive to l-serine, with IC_50_-values of ~5 and 10 mM, respectively. On the other hand, the Type I PGDH from *M. tuberculosis* has an IC_50_ for l-serine of ~30 μM, but only in the presence of phosphate ion (50–100 mM). In the absence of phosphate, the IC_50_ is in the low mM range (Xu and Grant, 2014) similar to that for *B. subtilis* and *C. glutamicum*. The available evidence shows that l-serine will bind to the ACT domain in the absence of phosphate ion but it is the interaction of phosphate at the ASB domain that allows l-serine to inhibit activity in the micromolar range (Burton et al., 2009b; Xu and Grant, 2014). However, the phosphate dependent sensitivity to l-serine does not occur with all mycobacteria (Xu et al., 2015) although it seems to be consistent among pathogenic mycobacteria to the extent that it has been studied. PGDH from all mammalian sources that have been studied show no sensitivity to serine at all, even though they contain ACT domains (Achouri et al., 1997; Dey et al., 2008; Grant, 2012). In these cases, the ACT domain residues are altered so that they no longer bind serine (see Figure 3). The classical feedback regulation by l-serine occurs with the Type II PGDH from *E. coli* which has an IC_50_ for l-serine of 2–10 μM, depending on the buffer used in the assay (Grant et al., 2000a, 2005). Since Type III PGDH enzymes do not contain an ACT domain, they are not feedback regulated by l-serine.

*E. coli* PGDH contains a Gly–Gly sequence (Gly 336–Gly 337 corresponding to positions 397–398 in Figure 3) between the substrate binding domain and the regulatory domain that acts like a hinge and plays a role in the conformational change that takes place upon serine binding and that is responsible for inhibition of activity (Grant, 2011). A similar sequence is found in *M. tuberculosis* PGDH (Gly 316–Gly 317–Gly 318, corresponding to positions 389–391 in Figure 3) but not in human PGDH. The absence of this hinge region in human PGDH is a contributing factor to its lack of feedback regulation by l-serine.

## Alternate Substrates

**Figure 11:**
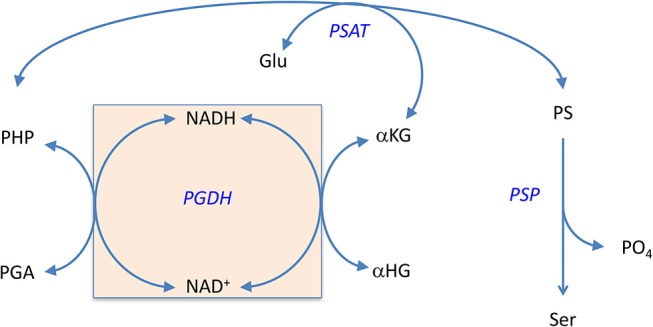
The self-sustaining cycle that regenerates coenzyme bound to PGDH during biosynthesis of l-serine in *E. coli*. The NADH that is produced during the conversion of PGA to PHP is converted back to NAD^+^ *in situ* by conversion of αKG to αHG. The αKG is formed from glutamate by the second enzyme in the pathway, PSAT. Reprinted from Grant (2018) with permission from the American Chemical Society.

In 1996, Zhao and Winkler discovered that *E. coli* PGDH could use α-KG as a substrate in the reverse direction in place of PHP (Zhao and Winkler, 1996) to produce α-hydroxyglutarate (α-HG) with the concomitant oxidation of NADH. The ability to use αKG as a substrate has also been reported for *Pseudomonas stutzeri* (*ps*PGDH) (Zhang et al., 2017), *Pseudomonas aeruginosa* (*pa*PGDH) (Zhang et al., 2017), *Saccharomyces cerevisiae* (*sc*PGDH) (Becker-Kettern et al., 2016), and human PGDH (*hs*PGDH) (Fan et al., 2015). However, the level of activity of human PGDH for α-KG is relatively low with a *k*_cat_ of ~0.08 s^−1^ compared to that for *E. coli* PGDH of 33 s^−1^ (Fan et al., 2015). PGDH from *Mycobacterium tuberculosis* (*mt*PGDH) (Dey et al., 2005b) and *Rattus norvegicus* (Achouri et al., 1997) are reported to not use α-KG as a substrate. Zhang et al. (Zhang et al., 2017) have shown in *Pseudomonas* species, that the coupling of this reaction with d-2-hydroxyglutarate dehydrogenase can serve to drive l-serine synthesis. Grant (2018) has shown that in *E. coli*, there is a process that conserves coenzyme in the production of l-serine by utilizing an intrinsic cycle of NAD^+^/NADH interconversion coupled with the conversion of αKG to αHG. Interestingly, this cycle can be maintained *in vitro* by production of αKG by the second enzyme in the pathway, PSAT, and does not require any additional enzymes (Figure 11). However, there is probably an ample pool of αKG available *in vivo* so that this is not strictly required. Note also that the kinetic mechanism shown in Figure S4 is for the reverse direction where NADH can displace NAD^+^. Therefore, the NADH conversion cycle is likely not functional in this direction.

No other compounds have been found to display significant activity as substrates. However, several NAD analogs have been demonstrated to be able to substitute for the coenzyme (Walsh and Sallach, 1965; Sugimoto and Pizer, 1968b; Rosenblum and Sallach, 1970; Winicov and Pizer, 1974; Unterlass et al., 2017). These include thionicotinamide adenine dinucleotide in wheat germ and human PGDH, 3-acetylpyridine adenine dinucleotide in wheat germ, *E. coli*, chicken liver, and human PGDH, 3-acetylpyridine deamino adenine dinucleotide in wheat germ and chicken liver PGDH, deamino nicotinamide adenine dinucleotide in *E. coli* and chicken liver PGDH, and 3-pyridinealdehyde adenine dinucleotide in chicken liver and human PGDH. Note, however, that not all coenzyme analogs have necessarily been tested with each species. In all cases, NADP^+^ displays < 10% of the activity of NAD^+^.

## Mechanism

Only *E. coli* (Grant et al., 2003, 2004; Burton et al., 2008, 2009a; Grant, 2018) and *M. tuberculosis* (Grant et al., 1999c; Dey et al., 2005a,b, 2008; Burton et al., 2007, 2009b; Xu and Grant, 2014; Xu et al., 2015) PGDHs have been studied extensively with regard to mechanism. A previous review (Grant, 2012) summarized the findings of these studies in detail so they will only be briefly summarized here.

### *E. Coli* PGDH

The mechanism of *E. coli* PGDH was studied in the reverse direction using α-KG and exhibits an ordered Bi Bi mechanism where NADH must be present before substrate binds (Burton et al., 2008). The *k*_cat_ of *E. coli* PGDH in the reverse direction is relatively slow at 7 s^−1^ per subunit with PHP as substrate. In the forward direction, the *k*_cat_ is ~0.6 s^−1^ in an uncoupled reaction with PGA as substrate. It has not been measured in the forward direction by coupling it to PSAT and monitoring the reduction of NAD^+^ because of the rapid regeneration of NAD^+^ from NADH described above (Figure 11). *E. coli* PGDH is isolated with tightly bound NADH and, for a long time, it was thought that only NADH was tightly bound to the resting enzyme. However, recent evidence (Grant, 2018) has shown that NAD^+^ is also rather tightly bound to the enzyme although it can be displaced by NADH. Both coenzyme forms appear to remain bound to the enzyme during continuous turnover when coupled to PSAT and participate in a cycle where they are continuously regenerated *in situ* (Grant, 2018). The sites that lie across the nucleotide binding domain interface appear to operate in a “flip-flop” manner (Figure S5), with each site trading places functionally after each turnover (Grant et al., 2003, 2004; Grant, 2018).

When enzyme catalysis is studied in the reverse direction with enzyme in which all of the NADH has been converted to NAD^+^, there are two kinetically distinguishable NADH binding phenomena (Burton et al., 2008, Figure S4). One where coenzyme is very tightly bound and induces a conformational change that increases the dissociation constant for coenzyme even more, and one that is less tightly bound (Burton et al., 2008). The former, found in the resting enzyme, can turn over in the presence of substrate but the latter is the one that is functional during continuous turnover. That is, the conformational change that occurs with the binding of NADH in the absence of substrate does not occur to an appreciable extent during continuous turnover. Note that these “sites” are distinguished kinetically rather than positionally. Also note that this was determined for *E. coli* PGDH and is likely not the same for human and *M. tuberculosis* PGDH.

A type of half-of-the-sites activity appears to be functional for inhibition of activity by serine binding (Grant et al., 2004). Although all four effector sites in the ACT domains eventually bind serine, only two, on opposite sides of the nucleotide binding domain, need to be occupied for optimal inhibition. Binding to these two sites displays positive cooperativity. Binding of serine to the last two sites is weaker due to negative cooperativity across the ACT domain interfaces.

The catalytic activity of *E. coli* PGDH can be inhibited in a reversible manner by cross-linking adjacent regulatory (ACT) domains with a disulfide bridge (Al-Rabiee et al., 1996a). Furthermore, this inhibition can be completely reversed by reduction of the bridge with dithiothreitol. This suggests that the regulatory domains move in some manner relative to each other during the transition from active to inhibited state. The disulfide bridge appears to mimic the binding of inhibitor since l-serine binds across the regulatory domain interface linking the two domains. Therefore, the mechanism of inhibition is one where serine binding eliminates a conformational change resulting from substrate binding and forms a dead-end quaternary complex consisting of enzyme, coenzyme, substrate, and effector (Burton et al., 2009a). Thus, the mechanism is a V-type that results in the reduction of active species rather than in a graded modulation of the velocity of the active enzyme.

Tryptophan 139 (position 187 in Figure 3), which sits at the bottom of the catalytic site of the adjacent subunit, plays a critical role in the oligomeric state of the enzyme as well as the integrity of the catalytic site (Grant et al., 2000b). When it is converted to a glycine residue, the enzyme dissociates into dimers and is 600-fold less active. However, it is just as sensitive to inhibition by l-serine as the native enzyme, although the cooperativity of inhibition is lost. It is quite surprising that a single residue can have such a drastic effect on the oligomerization state given that there is extensive subunit-subunit contact in this region, but this result demonstrates its critical importance. That this tryptophan is conserved in all other tetrameric PGDHs, suggests it plays a similar role in their catalytic and structural integrity.

Other than l-serine, which is a very potent inhibitor of *E. coli* PGDH, no other natural inhibitors have been identified. However, several heterocyclic organic inhibitors have been reported from studies involving predicted allosteric sites (Qi et al., 2012; Wang et al., 2014).

### *M. tuberculosis* PGDH

The *k*_cat_ of 600 s^−1^ per subunit in the reverse direction for *M. tuberculosis* PGDH is much faster than that of *E. coli* PGDH. However, when coupled to PSAT in the forward direction, it is only 1.4 and 3 s^−1^ at pH 7.0 in MOPS and phosphate buffer, respectively. The kinetic mechanism is also ordered Bi Bi, but in this case, substrate binds before coenzyme. Although the order was determined with a reverse direction assay, it is consistent with the observation that the *E. coli* enzyme crystallizes with coenzyme and shows strong affinity for 5′-AMP Sepharose, an NAD analog, while the *M. tuberculosis* enzyme does not crystalize with coenzyme bound to the active site and has no affinity for 5′-AMP Sepharose. Interestingly, pre-steady state analysis showed that NADH could in fact bind to the enzyme in the absence of substrate but that the binding constants were too slow to account for the catalytic reaction (Dey et al., 2008, Figure S6). Subsequent investigation (Burton et al., 2009b; Grant, 2012) indicated that NADH bound at or near the ASB site and reduced the amount of substrate inhibition due to substrate interaction at the ASB site. It is not known what the exact nature of the interaction of NADH with the ASB site is, but it does not have an inhibitory effect on the rate of catalysis. The dissociation constants for substrate binding to the catalytic and ASB sites are 0.09 and 8 μM, respectively.

Other than l-serine, which effectively inhibits *M. tuberculosis* PGDH only in the presence of substantial concentrations of phosphate ion, no other inhibitors, natural or synthetic, have been identified for PGDH from mycobacteria. However, our unpublished work has demonstrated that CBR5884, a potent inhibitor of human PGDH (Mullarky et al., 2016), does not inhibit *M. tuberculosis* PGDH. This limited evidence suggests that specific inhibitors could be found for PGDH from different species, and therefore, in light of the essential metabolic role of l-serine in metabolism, this is an area in need of further exploration.

### Human PGDH

No detailed studies of the mechanism of human PGDH have been reported but the kinetic constants for human PGDH are similar to those for *M. tuberculosis* PGDH when coupled to PSAT, with the exception that the *K*_m_ for NAD^+^ is ~8-fold higher for the human enzyme. Like *E. coli* PGDH, the enzyme appears to contain bound cofactor when isolated and the binding order appears to be coenzyme before substrate (Unterlass et al., 2017). Although the human enzyme has been reported to slowly convert αKG to αHG (Fan et al., 2015), the measured rate of conversion is very slow (Fan et al., 2015) and it does not sustain a coenzyme conservation cycle like that reported for *E. coli* PGDH (Grant, 2018). As mentioned earlier, while human PGDH is a Type 1 enzyme, it is not regulated by l-serine. In fact, human PGDH, as well as other mammalian PGDHs, have not been found to be regulated by any small molecule. Therefore, any regulation of human PGDH occurs either at transcription or translation or in the case of genetic mutations of amino acid residues as described below.

## Congenital Defects Associated With the Lack of PGDH in Mammals

Serine deficiency disorders, which are caused by defects in the pathway leading to the synthesis of l-serine, were first reported in 1996 (Jaeken et al., 1996) and are mostly neurological in nature (Yoshida et al., 2004; Furuya, 2008; van der Crabben et al., 2013; El-Hattab et al., 2016). Although defects in the expression or catalytic activity of any of the three biosynthetic enzymes in the pathway can lead to l-serine deficiency (Tabatabaie et al., 2010; van der Crabben et al., 2013), those related to PGDH deficiency are the most common. The important role of PGDH was shown with a knock-out mouse model. The phenotype was associated with embryonic lethality and clearly demonstrated the l-serine biosynthetic pathway was critical (Yoshida et al., 2004).

That l-serine synthesized in the brain was the source of d-serine in the mature brain was shown using a conditional knock-out of PGDH in the brain that bypassed the embryonic lethal phenotype resulting from systemic deletion (Yang et al., 2010). The study found that both l-serine and d-serine levels were significantly decreased in the cerebral cortex and hippocampus without altering levels of SR and NMDA receptor subunits. The study concluded that “in mature neuronal circuits, l-serine availability determines the rate of d-serine synthesis in the forebrain and controls NMDA receptor function at least in the hippocampus.”

Infantile, juvenile, and adult onset phenotypes have all been reported for PGDH deficiency (Tabatabaie et al., 2010; van der Crabben et al., 2013). In the infantile phenotype, damage to the brain has already occurred prior to birth and usually manifests itself with congenital microcephaly, intractable seizures, and severe psychomotor defects. In general, oral supplementation with l-serine is very effective in reducing seizures but has little effect on psychomotor function. The juvenile phenotype was found in two siblings and traced to a single amino acid mutation. These patients first showed symptoms after 5 and 9 years of age and were not diagnosed as such until they were teenagers. The symptoms were much milder than in the infantile phenotype, consisting of absence seizures and moderate developmental delay without microcephaly. Both responded well to oral supplementation with l-serine. The adult phenotype in a single individual consisted of congenital cataracts, mental retardation in childhood, and progressive polyneuropathy as an adult.

One mutation, in particular, has been found as a common cause of PGDH deficiency, having been found in at least seven different individuals (Tabatabaie et al., 2010). This mutation, Val490Met, is found in the regulatory or ACT domain of PGDH. It is not known how it results in PGDH deficiency and the literature contains conflicting conclusions. One group (Pind et al., 2002) reports that the mutation results in a decrease in expression and an increase in degradation of PGDH, while another group (Tabatabaie et al., 2009) concluded that the mutation was without effect on expression and degradation, but rather produced an enzyme with low residual activity. It is interesting to note that the mutation is in the ACT domain and if the human structure is the same as that reported for PGDH from *M. tuberculosis*, the side chain of residue 490 would be found at the subunit interface between the ASB/ACT domains from adjacent subunits. Although far from the catalytic site, this mutation could result in an interference of structural integrity of the oligomer that could result in reduced activity and enhanced susceptibility of enzyme to degradation, which could be consistent with both literature reports.

## PGDH and Cancer

In 1970, Davis et al. (1970) published the first indication that changes in serine biosynthesis may be related to cancer. This work showed that PGDH activity was greater in rat hepatoma cell lines compared to normal liver cells, and correlated the fastest growth rate with the highest PGDH activity. Subsequent work (Snell, 1984) showed that this was true in many other tumors as well, and that among the pathway enzymes, tumor growth was most consistently correlated with an increase in PGDH activity. Renewed interest in the link between serine biosynthesis and cancer has occurred within the last 10 years or so with the observation that an increased level of expression of PGDH has been found in human cancers such as breast (Possemato et al., 2011), cervical (Zhang et al., 2015), glioma (Liu et al., 2013), melanoma (Ou et al., 2015), colon (Yoon et al., 2015), pancreatic (Zhiwang et al., 2018), liver (Shanshan et al., 2017), kidney (Yoshino et al., 2017), and others as well. Based on multiple lines of evidence (Mattaini et al., 2016), the phosphorylated pathway of serine biosynthesis, which utilizes PGDH, is the sole source of serine synthesis in non-photosynthetic organisms, including humans. In most cases, tumor cell proliferation is associated with increased levels of PGDH and decreased cell proliferation is seen when PGDH is knocked out, even though exogenous l-serine is supplied. In addition, inactive PGDH, due to site-specific mutation, cannot support proliferation of PGDH-dependent cells (Mattaini et al., 2015). It has also been shown that increased levels of PGDH synthesis, as well as the other serine biosynthetic enzymes, correlates with patient survival outcome and may be used as a prognostic factor for some cancers (Antonov et al., 2014). In some cases, extracellular serine seems sufficient to promote tumor cell proliferation, whereas in other cases, extracellular serine is not able to support cell proliferation when PGDH is absent. For instance, adding excess serine to the growth medium of PGDH knockdown human breast cancer cells was not able to rescue cell proliferation (Possemato et al., 2011; Chen et al., 2013). This suggests that flux through the serine biosynthetic pathway is providing something other than just a source of serine. In those cases where extracellular serine seems sufficient, additional reactions may promote flux through the pathway (Figure S7). It is still unknown just what the link is, but the requirement for pathway flux suggests that increased levels of the pathway intermediates, such as PHP or phosphoserine, may be critical. This has become a very active area of investigation in recent years and more detailed information on the link between cancer and PGDH can be found in several reviews (Luo, 2011; Mullarky et al., 2011; Zogg, 2014; Mattaini et al., 2016).

As a result of the correlation between high PGDH expression levels and cancer, there has been interest in finding inhibitors of the enzyme activity as potential starting points for drug development. Several recent articles have reported advancements in this area (Mullarky et al., 2016; Pacold et al., 2016; Wang et al., 2016; Ravez et al., 2017; Unterlass et al., 2018).

## Summary and Prospective

d-3-Phosphoglycerate dehydrogenase catalyzes the same reaction in all known organisms where it is found. That is, the NAD^+^/NADH dependent interconversion of d-phosphoglycerate and PHP. However, there are significant differences among organisms as to how or whether PGDH activity is regulated by other factors. In all organisms where it has been studied, the equilibrium of the PGDH reaction lies far in the direction away from serine synthesis. Therefore, the downstream enzymes play a large role in keeping the pathway flux moving in the direction of serine synthesis by depleting PHP and then phosphoserine. The last reaction of the pathway is irreversible, so once l-serine is produced it cannot be converted back to PS by simple reversal of the pathway. This presumably provides a relatively stable pool of l-serine that is available for conversion into other metabolites like d-serine. In *E. coli*, for example, there exists a very sensitive regulation of PGDH activity by l-serine that provides for fine tuning of the l-serine pool. A similar feedback mechanism exists in *M. tuberculosis* PGDH, except that the enzyme's sensitivity to l-serine is modulated by the available phosphate ion content. Yet, in other organisms, such as *C. glutamicum* and some plants, the serine sensitivity is as much as three orders of magnitude less. In humans, inhibition of PGDH by l-serine does not occur.

Very little is known about the regulation of PGDH or the serine biosynthetic pathway itself in mammalian cells or organisms, including *H. sapiens* especially. The attraction to the bacterial enzymes for detailed investigation may be partly due to the perception that the bacterial enzymes are more interesting from an enzymological point of view in that the human enzyme does not appear to be regulated by an effector molecule such as l-serine. This lack of feedback control in human PGDH may provide for a relatively large pool of available serine for conversion into other metabolites as well as protein synthesis. Although the literature is very sparse in this regard, it does not seem that the other enzymes in the serine biosynthetic pathway in mammals display any significant level of regulation by small molecule effectors. Since the available literature clearly points to the availability of l-serine as being critical to the synthesis of d-serine, this would seem to be a particularly relevant area of investigation in regards to the function and physiology of the NMDA receptor.

One very important facet of the study of the human enzyme that is missing is a complete picture of its structure which would aid in any conclusions about its mechanism that might be made relative to the *M. tuberculosis* enzyme, another Type I PGDH. The availability of a complete structure of human PGDH will also be necessary in the evaluation and fine tuning of inhibitors that may eventually be developed into drugs targeting PGDH activity in malignant cells and tumors.

From a physiological viewpoint, a thorough analysis of the flux of the serine biosynthetic pathway in mammalian astrocytes, in response to different stimuli, will be useful. An even broader question in this respect concerns the interplay between glycolysis, gluconeogenesis, and serine synthesis. If the l-serine biosynthetic pathway in mammals lacks any type of specific regulatory mechanisms to control the production of l-serine, what factors govern consumption of the common metabolite, PGA? Asked another way, what keeps the serine pathway in these organisms from consuming an excess of PGA and adversely affecting energy production from glucose?

The studies conducted with malignant mammalian cells demonstrate that the expression of PGDH can be modulated, but the precise factors leading to changes in expression levels are not well-understood. This would also seem to be an area requiring further investigation that may be relevant not only to cancer but to neurological function as well.

## Author Contributions

The author confirms being the sole contributor of this work and has approved it for publication.

### Conflict of interest statement

The author declares that the research was conducted in the absence of any commercial or financial relationships that could be construed as a potential conflict of interest.
