# Supplementary material for: D-3-Phosphoglycerate Dehydrogenase
Source: Front Mol Biosci. 2018 Dec 13;5:110. doi: 10.3389/fmolb.2018.00110 (PMC6300728; doi:10.3389/fmolb.2018.00110)
Supplement: Supplementary file 1 [file Data_Sheet_1.PDF]

Supplementary Material

D-3-Phosphoglycerate Dehydrogenase

Gregory A. Grant

Departments of Developmental Biology and Medicine, Washington University School of Medicine, 660 S. Euclid Avenue, Box 8103, St. Louis, Missouri 63110.

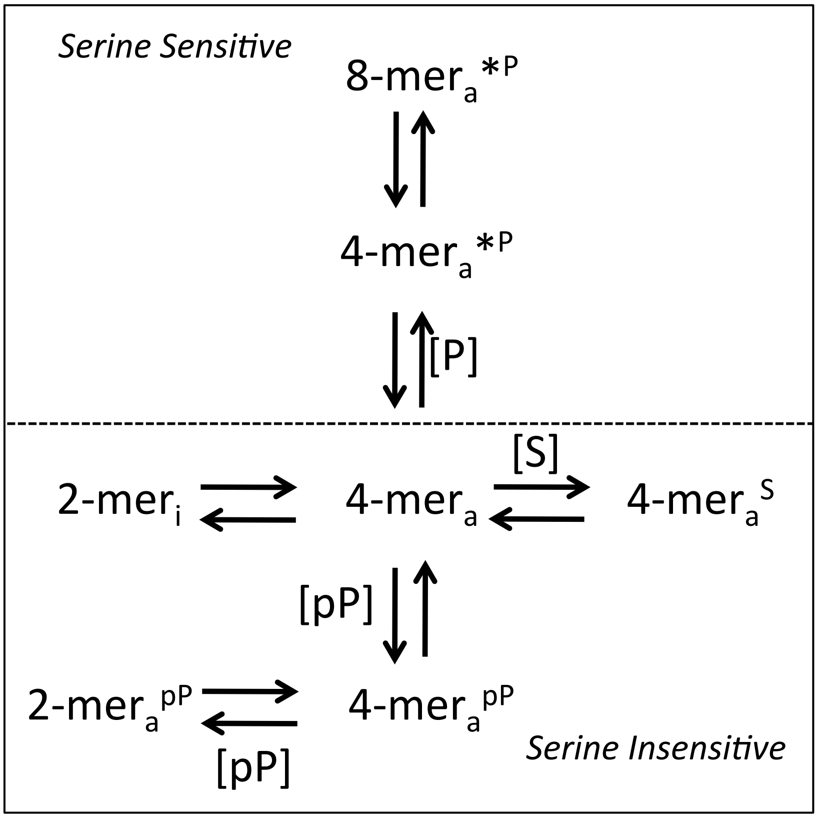

Figure S1. The oligomeric forms of *M. tuberculosis* PGDH as a function of the presence of phosphate and pyrophosphate. Reprinted from Biochemistry 53(26), Xu, X. L., and Grant, G. A. Regulation of *Mycobacterium tuberculosis* D-3-phosphoglycerate dehydrogenase by phosphate-modulated quaternary structure dynamics and a potential role for polyphosphate in enzyme regulation, 4239-4249, with permission from the American Chemical Society.

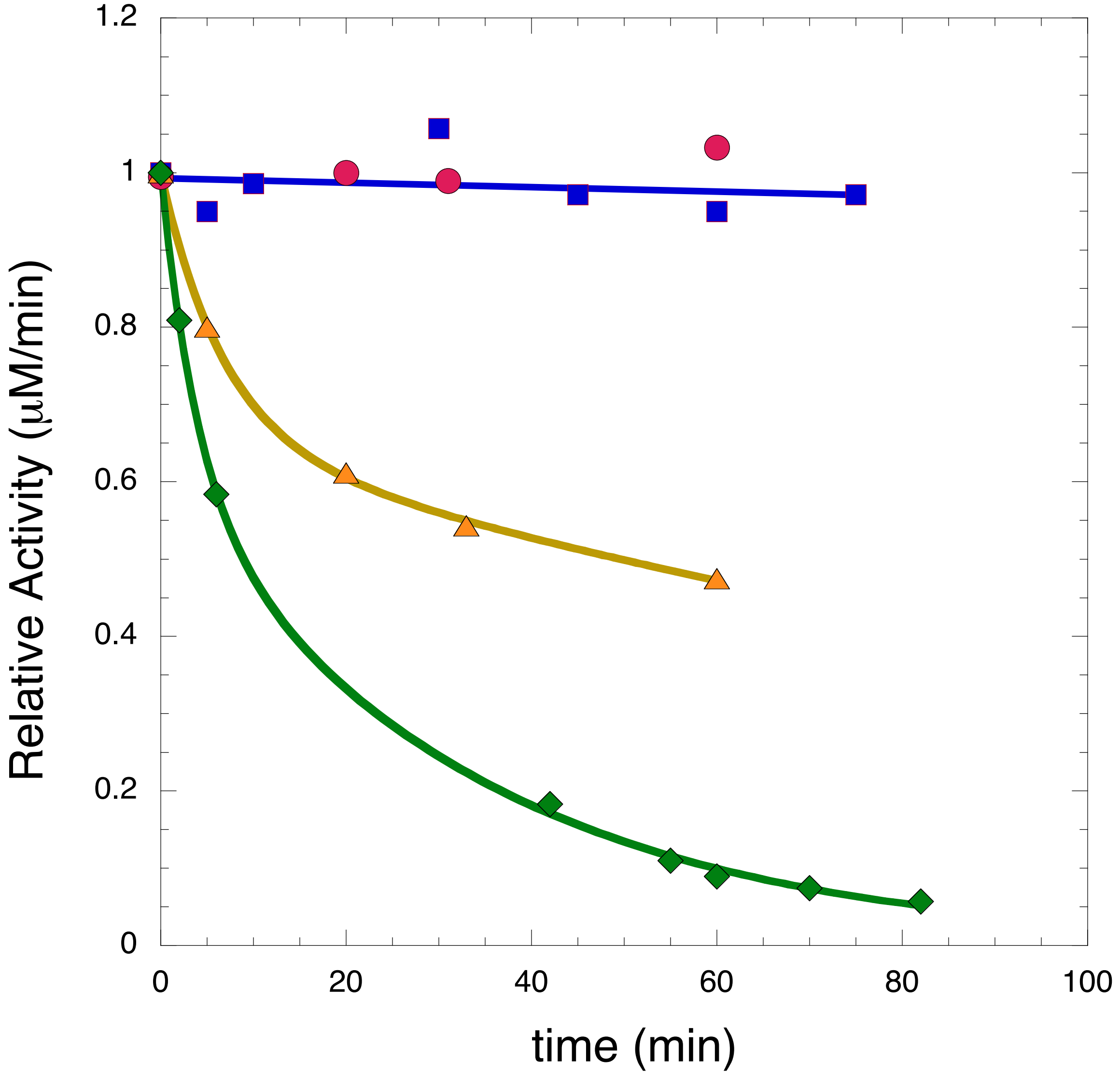

Figure S2. The effect of dilution and dependency on phosphate ion as a function of time on the activity of *M. tuberculosis* PGDH. The enzyme is diluted at time zero from 200 mM KPO_4 buffer, pH 7.5. 1/500 dilution in 200 mM KPO_4 buffer, pH 7.5 (■), 1/10 dilution in 20 mM KPO_4 buffer, pH 7.5 (●), 1/100 dilution in 20 mM KPO_4 buffer, pH 7.5 (▲), 1/500 dilution in 20 mM KPO_4 buffer, pH 7.5 (◆).

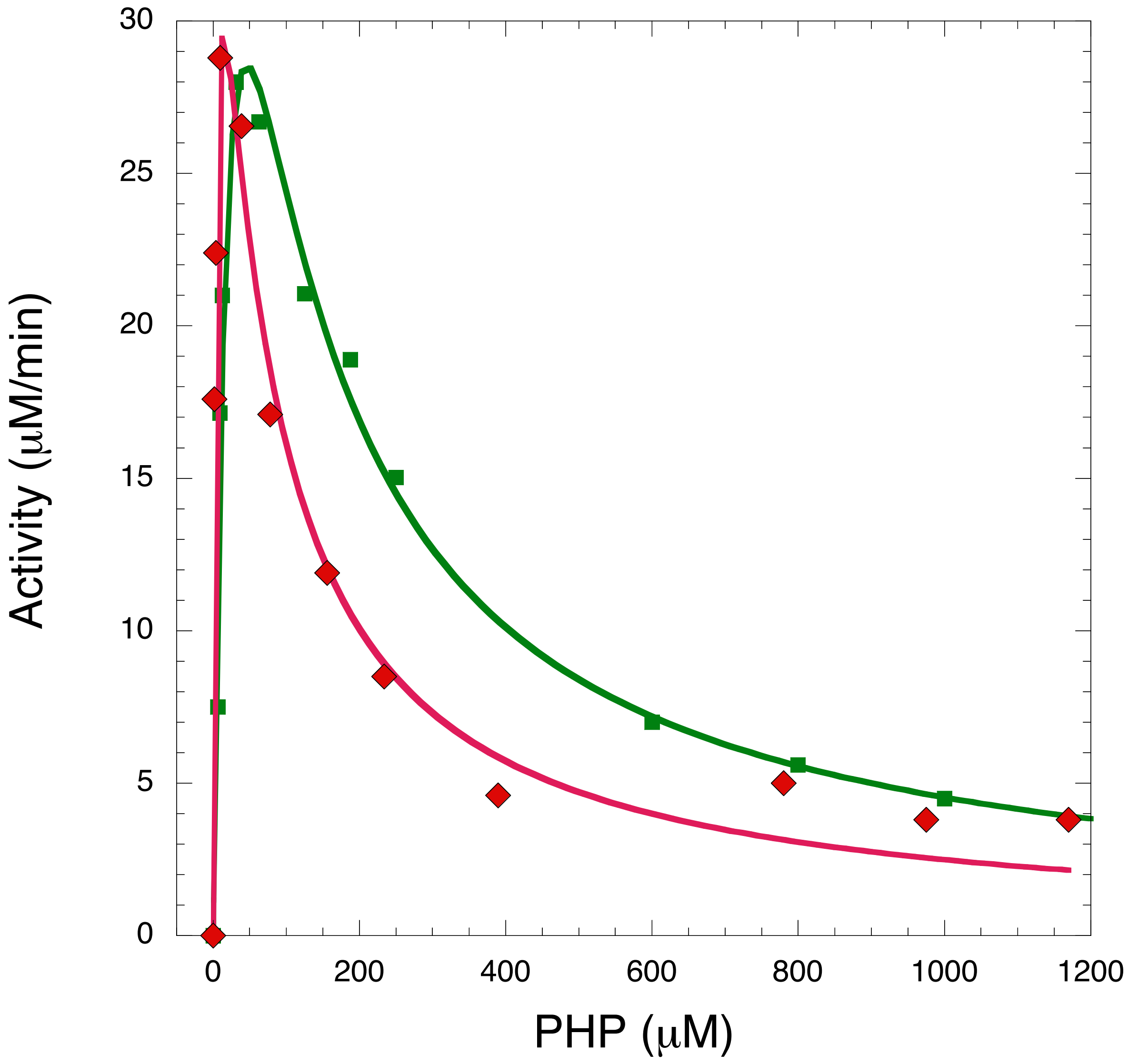

Figure S3. Plots of the catalytic activity of PGDH as a function of substrate concentration (PHP) in the reverse direction, towards PGA production, showing significant substrate inhibition for *M. tuberculosis* PGDH (green), and *H. sapiens* PGDH (red).

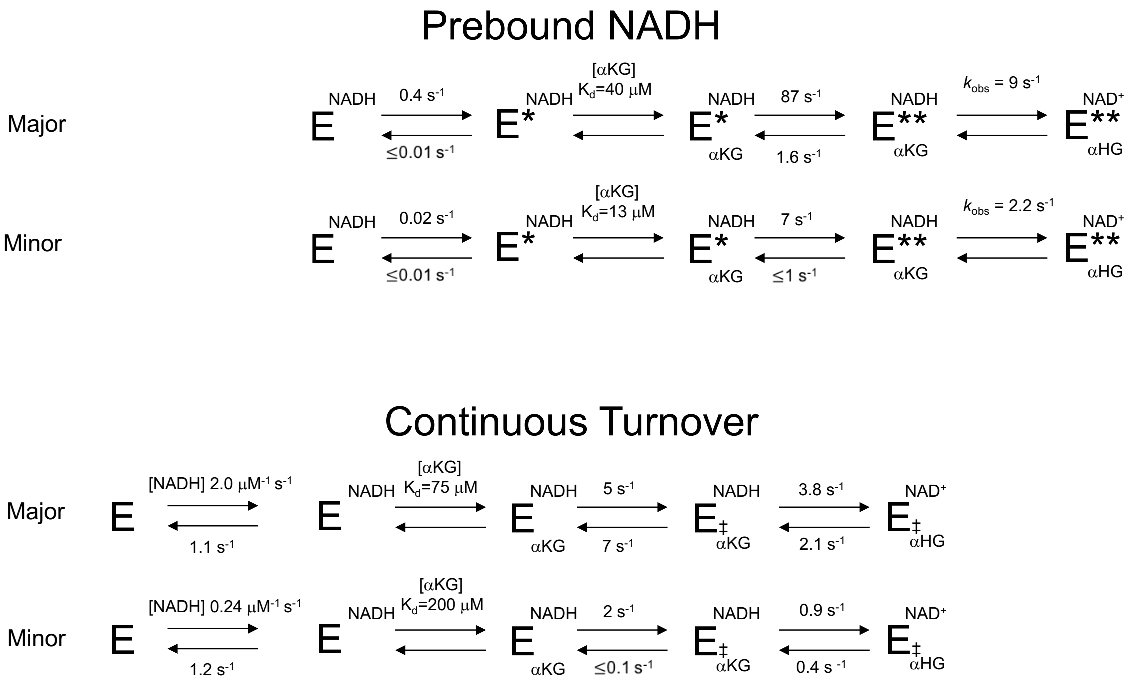

Figure S4. Pre-steady-state kinetics of *E. coli* PGDH analyzed in the reverse direction. When NADH is pre-bound to the enzyme (top), a single turnover step results when substrate (αKG) is added, followed by continuous turnover (bottom). The values are expressed as per subunit. In each case, the major path represents the activity of the enzyme in the predominate site while the minor path is for the other site that lies across the nucleotide binding domain interface. The terms E, E*, E**and E‡ designate different conformations of the enzyme as the reaction progresses. Superscripts and subscripts above and below the E symbol represent bound ligand. (Figure redrawn from reference 49).

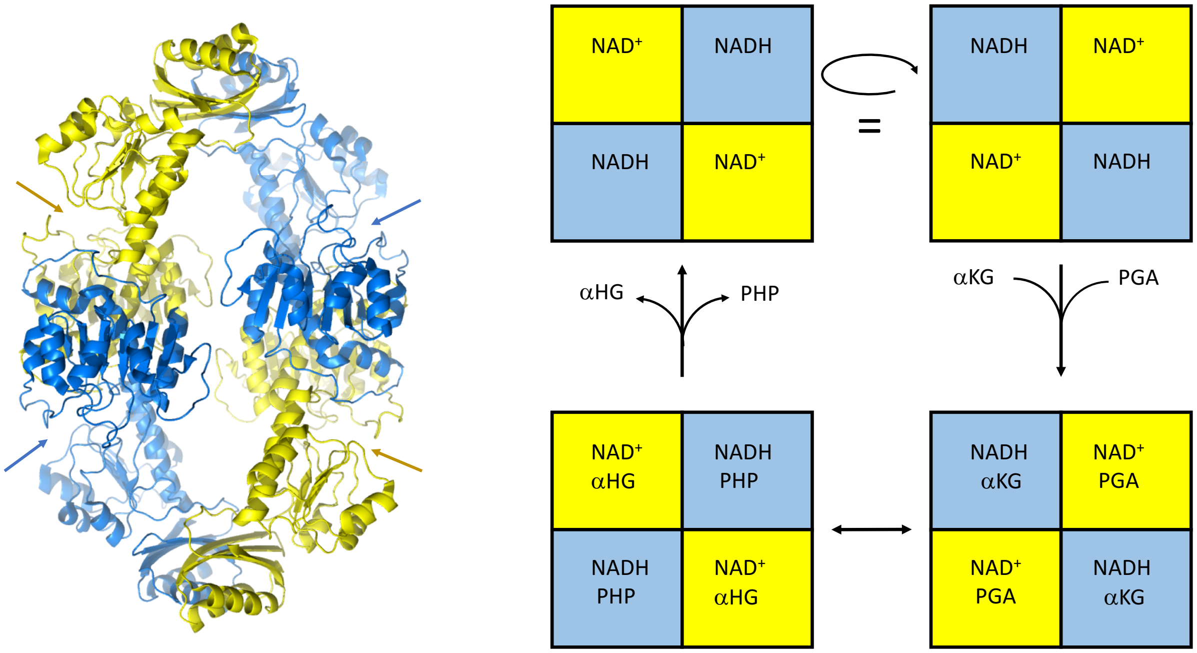

Figure S5. The *ec*PGDH tetramer is illustrated as a ribbon diagram (PDB: 1YBA) on the left with the arrows indicating the active site clefts. The subunits are colored alternately yellow and blue. On the right is a diagram depicting how the subunits may function in a flip-flop manner to simultaneously catalyze the conversion of the substrate pairs, NADH/αKG and NAD^+/PGA. Note that since the tetramer is symmetrical, the top two representations are equivalent and can be superimposed with a 180º rotation. Reprinted from Biochemistry 57, Grant, G. A. Elucidation of a self-sustaining cycle in *E. coli* L-serine biosynthesis that results in the conservation of the coenzyme, NAD^+, 1798-1806, with permission from the American Chemical Society.

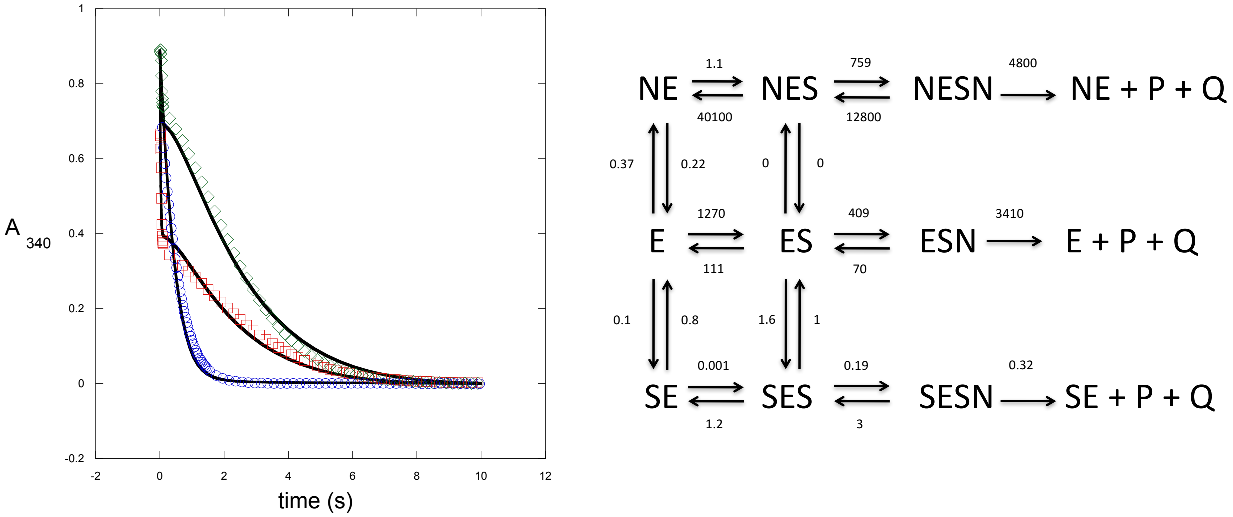

Figure S6. Pre-steady-state kinetics of *M. tuberculosis* PGDH monitored at 340 nm for the conversion of NADH to NAD^+. Kinetic constants were determined from a fit of the data to the model on the right using Global Kinetic Explorer from KinTek corporation. Data is represented by symbols and the fits are shown as solid lines. The rapid mixing protocols are enzyme mixed with PHP and NADH (red), enzyme pre-incubated with NADH and then mixed with PHP and NADH (blue), and enzyme pre-incubated with PHP and then mixed with PHP and NADH (green). In the model, E represents the enzyme, N is coenzyme, and S is substrate. When placed to the right of E it designates binding at the catalytic site, when place to the left of E, it designates binding at the effector site. Units for ligand binding are $\mu M^{-1}\ s^{-1}$ and all others are s^{-1}.

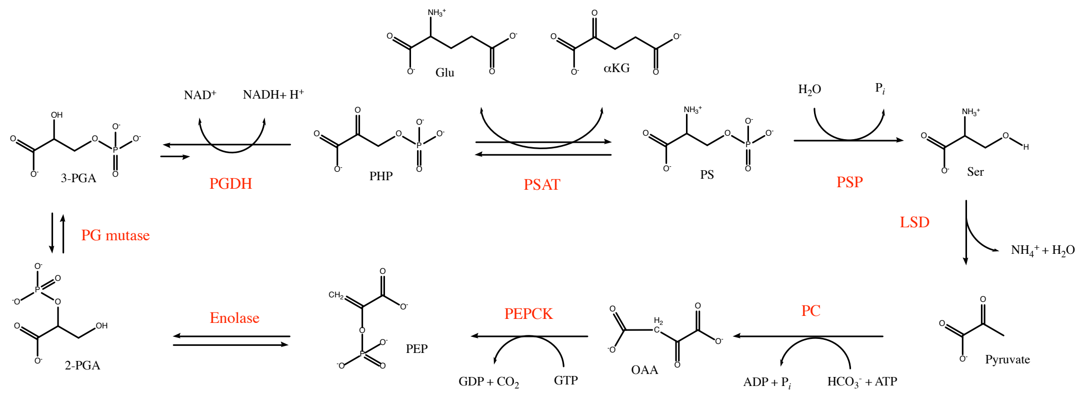

Figure S7. The production of serine by the serine biosynthetic pathway and its conversion back to D-3-phosphoglycerate by an abbreviated gluconeogenic pathway.
